# Cost-effectiveness of treatment sequences following first-line rituximab in relapsing-remitting multiple sclerosis: a Norwegian microsimulation study

**DOI:** 10.3389/fneur.2026.1783506

**Published:** 2026-03-18

**Authors:** Simone Huygens, Matthijs Versteegh, Pål Berg-Hansen, Stein Henry Bjelland, Trygve Holmøy, Øivind Torkildsen

**Affiliations:** 1 Huygens and Versteegh B.V., Zwijndrecht, Netherlands; 2 Department of Neurology, Oslo University Hospital, Oslo, Norway; 3 Vestfold Hospital Trust, Vestfold, Norway; 4 Akershus University Hospital, Akershus, Norway; 5 Institute of Clinical Medicine, University of Oslo, Oslo, Norway; 6 Neuro-SysMed, Department of Neurology, Haukeland University Hospital, Bergen, Norway; 7 Department of Clinical Medicine, University of Bergen, Bergen, Norway

**Keywords:** cost-effectiveness, multiple sclerosis, rituximab, switching behavior, treatment sequencing

## Abstract

**Background:**

The Norwegian guideline recommends highly effective disease-modifying therapies (DMTs) as the first line treatment for multiple sclerosis (MS), with rituximab preferred in clinical practice. While rituximab is effective, patients may discontinue due to side-effects or develop disease activity. Limited guidance exists on optimal switches following first line rituximab.

**Objectives:**

To estimate the costs and effects of different treatment sequences following first line rituximab in relapsing remitting MS patients in Norway.

**Design:**

A microsimulation model adapted to the Norwegian setting estimated outcomes for different treatment sequences following first line rituximab. Four neurologists were interviewed using a structured expert elicitation tool to inform switching behavior. The model allowed for three treatment lines after rituximab, with switches possible to fingolimod, ponesimod, cladribine tablets, or natalizumab.

**Methods:**

The model simulated all 32 possible treatment sequences and calculated costs per quality adjusted life year (QALY) according to Norwegian pharmacoeconomic guidelines.

**Results:**

Neurologists were more likely to switch patients with >1 relapse (78%) or relapse and disability progression (66%) versus one relapse (54%). The most cost-effective sequence after rituximab is cladribine tablets (line 2), ponesimod (line 3), and natalizumab (line 4). The probability that second line cladribine tablets are most cost-effective is >75%. Mean time on first line rituximab was 15.2 years. The model predicts that over lifetime 21% of patients would require a fourth line of treatment due to the cumulative effects of discontinuation and recurring disease activity triggering treatment switches.

**Conclusion:**

Patients on first line rituximab may require a treatment switch due to side-effect induced discontinuation or new disease activity. If a switch from rituximab to another DMT is the preferred clinical course of action, this study showed that it is most cost-effective to switch to cladribine tablets followed by ponesimod and natalizumab.

## Introduction

The off-label use of rituximab as a treatment for multiple sclerosis (MS) is common in Northern Europe, and the Norwegian guideline lists it as preferred first line anti-CD20 treatment, reserving the more costly anti-CD20s for whom rituximab is considered unsuitable (1). Rituximab has been demonstrated to be a very effective treatment, also in treatment of naïve patients (2). However, disease activity may still occur and some patients develop serious side effects. A recent Norwegian registry study identified, among data of 159 patients, 17% of new MRI lesions and 6.8% discontinuation at month 48 (3). This raises the question of the selection of a follow-up treatment for those who have some form of disease activity or intolerance to rituximab.

Selecting a follow-up treatment is also discussed in the Norwegian guideline: patients on highly effective (“høyeffektive”) treatment (anti-CD20, natalizumab, S1PR modulators or cladribine tablets, according to the guideline) who experience “troublesome” or “serious” side-effects can be switched to another highly effective treatment. For patients who experience new disease activity while on highly effective therapy, the threshold to switch is higher as there are fewer options available (1). Thus, the guideline acknowledges that side-effects and new disease activity may result in a treatment switch, but leaves room for heterogeneity in switching behavior between different neurologists.

Which sequence of treatments brings most health benefits for patients, in balance with the associated costs to the health care system, is a function of the efficacy of treatments, their costs, and the attitudes toward switching of prescribing neurologists. A previously developed cost-effectiveness model for The Netherlands (the ErasmusMC/iMTA MS treatment sequence model) integrates treatment efficacy in reducing relapses and disability progression derived from network meta-analyses with probabilities of switching patients under certain circumstances derived from neurologists (4–6). This model was recently updated to incorporate the impact of aging using data from the MS Base registry (7).

In this study, we used a structured expert elicitation tool to understand the likelihood Norwegian neurologists will switch patients under different circumstance of new disease activity, including uncertainty. We integrated these switch probabilities in a new version of the Erasmus MC/iMTA MS treatment sequence model that is adapted to Norway, using Norway-specific costs, quality of life, mortality rates and possible treatment patterns. We subsequently used the model to identify the most effective and most cost-effective treatment options after first line rituximab in Norway.

## Materials and methods

### Structured expert elicitation on prescription behavior

Two researchers [one female (SH), one male (MV), both with PhD degrees in health economics and 6 years of experience MS research experience] conducted structured expert elicitation interviews with four Norwegian MS neurologists (PH, SB, TH, ØT) using a structured questionnaire developed by the research team (see Supplementary material). The questionnaire was not formally validated or pilot tested but was informed by clinical expertise and prior use in two unpublished international studies. Neurologists were approached by the funder via e-mail, and all approached neurologists agreed to participate. All neurologists were male, experienced in MS treatment with DMTs, and had no prior relationship with the research team. They were informed of the research team’s goals prior to participation. All participants were interviewed once via video calls without recording; one researcher took notes during the interview. Neurologists did not review interview notes but reviewed the final manuscript outcomes and interpretation, serving as co-authors. The average interview duration was 45 min.

The “structured expert elicitation resources” (STEER) tool, developed by the University of York, was used to elicit quantitative information on Norwegian-specific treatment switch behavior (8, 9). We used the most frequently used fixed interval methods in structured expert elicitation called the “roulette” or “chips and bins” method (10). This method provides experts with a grid that divides the plausible range of their answer into intervals. Experts were then asked to create a histogram by allocating “chips” (probability units, e.g., 10 chips is worth 5%) among the different “bins” (intervals) (10). An example histogram is provided in Supplementary Figure 1. Responses that were not elicited with STEER were summarized, and consensus or majority answers determined model structure.

Eight quantitative questions were answered with this method. Six questions regarding switching behavior and two regarding stopping DMT treatment in patients with stable disease. The quantitative question regarding switching of DMTs was “What proportion of patients with disease activity (see definitions below) while on a highly or moderately effective DMT would you switch to another DMT?” This question was asked for three types of disease activity (relapse in the previous year, relapses in two subsequent years, or relapse and disability progression in previous year), for both patients on highly or moderately effective DMTs, resulting in six questions in total. Highly effective DMTs included cladribine tablets, natalizumab, anti-CD20s and S1PRs (in line with the Norwegian clinical guideline) and moderately effective DMTs included interferons, glatiramer, fumarates, and teriflunomide. The two questions regarding stopping DMT treatment in patients with stable disease were: “What is the age from which you would consider stopping treatment under stable disease?” and “What is the number of years a patients should have stable disease for you to consider stopping treatment?” The beliefs of the individual MS neurologists were pooled with each expert assigned equal weight. Beta distributions were fitted to the six questions on switching behavior, which were probabilities and thus naturally bounded between 0 and 1, and normal distributions were fitted to the two questions regarding stopping DMT treatment. Chips counts were converted to cumulative probabilities at bin boundaries and passed to the “fitdist” function of the SHELF R-library. The “fitdist” function minimizes the sum of squared differences between the elicited cumulative probabilities and the fitted Beta cumulative distribution function, using numerical optimization. In accordance with the SHELF standard methods bins without any chips were assigned half a chip. Goodness of fit was assessed by visual comparison of the plotted observed and fitted cumulative density functions of each expert for each individual question (32 plots, see Supplementary Figure 7). In addition, the individual experts and pooled probability density plots are provided as well as the pooled mean and standard deviation of the outcomes.

In addition to the quantitative questions for the STEER tool, the semi-structured interview consisted of open questions concerning the number of DMTs (lines of treatment) patients typically undergo over their lifetime given the DMTs that are currently available, use of DMTs in different treatment lines, and selection of DMTs when a switch is necessary.

#### Leave-one-out analysis

To assess the robustness of the pooled expert estimates, we conducted a leave-one-out sensitivity analysis for each of the eight elicitation questions. For each question, one expert was removed from the expert panel and the pooled mean was recalculated. We recorded the absolute and relative difference between the pooled mean of the leave-one-out analysis and the full analysis and identified the expert whose exclusion resulted in the maximum deviation. To assess whether any influential responses had impact on the model outcomes in deterministic analysis, we used the maximum leave-one-out differences as alternative inputs in a sensitivity analysis with the cost-effectiveness model and compared the results to the base-case estimates.

### Cost-effectiveness treatment sequence model

We estimated the costs and effects of different treatment sequences following first line rituximab in relapsing remitting MS patients in Norway with a Norwegian adaptation of the Erasmus MC/iMTA MS model (4–7, 11, 12). The adaptation followed the Norwegian cost-effectiveness guidelines (13). This model estimates benefits of treatments by combining relative efficacy of DMTs from clinical trial populations with background probabilities from registry data. In contrast to the previous versions of the Erasmus MC/iMTA MS model (4–6, 11, 12), the background probabilities in the most recent version were based on the transition probabilities for EDSS progression as published by the MS Base registry by Campbell et al. (7, 14). Model characteristics have been published elsewhere (4–7, 11, 12). The model was adapted to the Norwegian setting with Norwegian specific input parameters and informed by structured elicitation interviews with Norwegian MS neurologists. There were several structural changes compared to the original Erasmus MC/iMTA MS model (4–6, 11, 12) for the Norwegian adaptation: the maximum number of treatment lines was changed from five to four, rituximab was included as first line treatment option, and productivity costs were excluded as per Norwegian cost-effectiveness guidelines (13).

### Norwegian input parameters

#### Treatment sequences and probabilities to switch

The Norwegian version of the Erasmus MC/iMTA MS model included four lines of DMT treatment in total. It should be noted that not all patients in the cohort that enter the simulation model will receive all four treatment lines, as only those with disease activity or discontinuation due to adverse events will switch to the next treatment line. The first line DMT in the treatment sequence model was rituximab, which reflects the current clinical practice in Norway. Based on the input of the four MS neurologists we included natalizumab, cladribine tablets, fingolimod and ponesimod as potential DMTs in the subsequent treatment lines. Excluded from the model were alemtuzumab (very rarely used in clinical practice), ozanimod (not on Norwegian procurement list except for patients with RRMS and Crohn’s disease or colitis ulcerosa as comorbidity) and ocrelizumab/ofatumumab/ublituximab (not used after primary loss of response on rituximab). Switches between fingolimod and ponesimod were excluded as they have the same mode of action and switches to fingolimod or ponesimod after natalizumab were excluded due to the high risk of rebound disease, resulting in eight possible treatment sequences. Probabilities to switch were based on structured expert elicitation and are reported in the Results section.

#### Efficacy and discontinuation due to side effects with rituximab

Efficacy [in terms of annualized relapse rates and 24-week confirmed disability progression (CDP)] and discontinuation due to side effects of DMTs were based on previous network meta-analyses and assumed constant irrespective of their position in the DMT sequence (e.g., first- versus second-line) (6, 15). While relative risks are constant between lines, they are applied to time-variant background probabilities for relapses and disease progression, causing differential effect of treatments depending on their position in the pathway. Efficacy of rituximab regarding relapses was included in the network meta-analysis using relapse rate data from a phase 2 trial (RIT 1,000 mg) (16) and the RIFUND-MS trial (RIT 500 mg) (2). These trials did not provide the necessary data on CDP and discontinuation due to side effects. Instead, we assumed that the efficacy of rituximab to prevent disability progression was equal to ocrelizumab, an assumption confirmed during interviews with the clinical experts. The risk of discontinuation due to side effects on rituximab was estimated by multiplying the relative risk (RR) of rituximab versus cladribine tablets (RR = 0.32) observed in Norwegian registry data reported by Rød et al. (3) with the RR of discontinuation due to side effects of cladribine tablets versus placebo (RR = 0.83) in the meta-analysis of Liu et al. (15), resulting in an RR of rituximab versus placebo of 0.27 for discontinuation.

After first-line rituximab, we allow natalizumab, cladribine tablets, fingolimod and ponesimod as potential DMTs in the subsequent treatment lines. A wash-out period of 3 months is often recommended after discontinuing anti-CD20 therapy (17). Our model assumed that the treatment initiated after primary loss of response of first line rituximab would be started after this wash-out period but within at maximum 12 months since the last dosage. For natalizumab, the risk of progressive multifocal leukoencephalopathy (PML) was included as an overall risk across patients on natalizumab. The annual probability of PML when using natalizumab was based on results of the period safety update report of natalizumab of the manufacturer as reported in the Dutch reimbursement dossier of natalizumab and was dependent on the time since start of treatment (Year 1: 0.00005, Year 2: 0.00063, Year 3: 0.00184, Year 4: 0.00236, Year 5: 0.00237, Year 6 and onwards: 0.00195) (18).

#### Costs

Acquisition costs of DMTs were based on Norwegian list prices and dosing schedules (Table 1). The associated costs of administration of DMTs, monitoring and patient travel costs were based on a report of the Norwegian Institute of Public Health (Table 2) (19). The annual costs of healthcare consumption per EDSS score class and relapses were based on Svendsen et al. (20) (Table 3). The reported values were corrected for costs of DMTs and relapses, because these costs were included separately in the model. The standard errors of the costs were not reported, therefore we assumed the standard errors to be equal to the mean estimate based on the mean values and standard errors of healthcare costs per EDSS score in nine other countries (21). The healthcare costs associated with switching DMT treatment were assumed to be equal to the diagnosis-related costs group (DRG 901D1) of an outpatient consultation regarding MS. There was no data on the amount of informal care used by Norwegian MS patients, therefore we used Dutch estimates and calculated costs by multiplying the Dutch resource use with the Norwegian unit cost of informal care (328 NOK/h) (22). Costs of adverse events were only included for PML for patients on natalizumab. There was no Norwegian estimate available, therefore a Dutch estimate of these costs was converted to NOK (NOK 368,071) (18). All costs were corrected for inflation to 2024 based on the Statistics Norway (SSB) consumer price index.

**Table 1:** Acquisition costs in NOK of DMTs based on Norwegian list prices and dosing schedules.

DMT	Year 1	Year 2	Year ≥3
CLA	254,903	254,903	–
FIN	204,077	204,077	204,077
PON	202,192	202,192	202,192
NAT	173,927	173,927	173,927
RIT	15,791	10,528	10,528

CLA, cladribine tablets; FIN, fingolimod; PON, ponesimod; NAT, natalizumab; RIT, rituximab; DMT, disease modyfing treatment; NOK, Norwegian krone.

**Table 2:** Administration, monitoring, and travel costs in NOK of DMTs based on Norwegian list prices and dosing schedules.

DMT	Year 1	Year 2	Year ≥3
CLA	9,700	6,119	5,973
FIN	18,882	6,119	6,119
PON	13,102	6,119	6,119
NAT	67,982	61,688	61,688
RIT	20,024	13,742	13,742

CLA, cladribine tablets; FIN, fingolimod; PON, ponesimod; NAT, natalizumab; RIT, rituximab; DMT, disease modyfing treatment; NOK, Norwegian krone. Without MRI and medical consultations costs and corrected for inflation to 2024. For PON: Assumption equal to FIN (excluding ECG observation at first time administration).

**Table 3:** Acquisition costs in NOK of healthcare and informal care consumption by EDSS score class.

Health state	Healthcare consumption	Informal care consumption
EDSS 0–3	139,352	22,451
EDSS 4–6	448,430	101,920
EDSS 7–9	972,609	230,256
Relapse	34,359	
Switch DMTs	3,030	

EDSS, Expanded Disability Status Scale; DMT, disease modyfing treatment; NOK, Norwegian krone. Healthcare consumption including MRI and medical consultations.

#### Quality-adjusted life years

Norwegian utilities by EDSS score were derived from Svendsen et al. (20). Since no utility value was reported for EDSS score 0, we estimated this using quadratic interpolation (see Table 4). The disutility for relapse was 0.071 (23). These utilities were combined with age and sex specific Norwegian background mortality from 2023 derived from the Human Mortality Database and excess mortality hazard ratios by EDSS score (24, 25) to estimate QALYs. The annual disutility of PML was derived from the Dutch reimbursement dossier for natalizumab and was based on expert opinion (0.13) (18).

**Table 4:** Utilities by EDSS score.

EDSS score	Utility
0	0.857
1–3	0.800
4–6	0.617
7–9	0.211

EDSS, Expanded Disability Status Scale.

### Willingness-to-pay threshold

The willingness-to-pay threshold depends on the severity of the disease expressed in absolute shortfall. The quality-adjusted life expectancy of patients with MS was 12.7 years according to our model. The quality-adjusted life expectancy of the general population was 36.2 years, resulting in an absolute shortfall of 23.5 years which corresponded with a willingness-to-pay threshold of 825,000 NOK (approximately 71.000 Euros and 83.000 US dollars).

### Cost-effectiveness analyses

The model simulated 10,000 patients per DMT sequence and the outcomes represent the average costs and QALYs of this virtual population. We performed a fully incremental analysis and calculated the net health benefit (NHB) for each treatment sequence. NHB reflects the cost-effectiveness of a treatment sequence and expresses the net benefit of a treatment in terms of QALYs, adjusted for the cost given the value of a QALY (NHB = QALYs − (costs/cost-per-QALY threshold)). For comparisons across the treatment sequences, all model parameters were varied simultaneously by sampling from their distributions in probabilistic sensitivity analyses (PSA). We conducted the PSAs with 500 sampling iterations while sampling 1,000 patients per treatment sequence.

## Results

### Structured expert elicitation on prescription behavior

**Figure 1:**
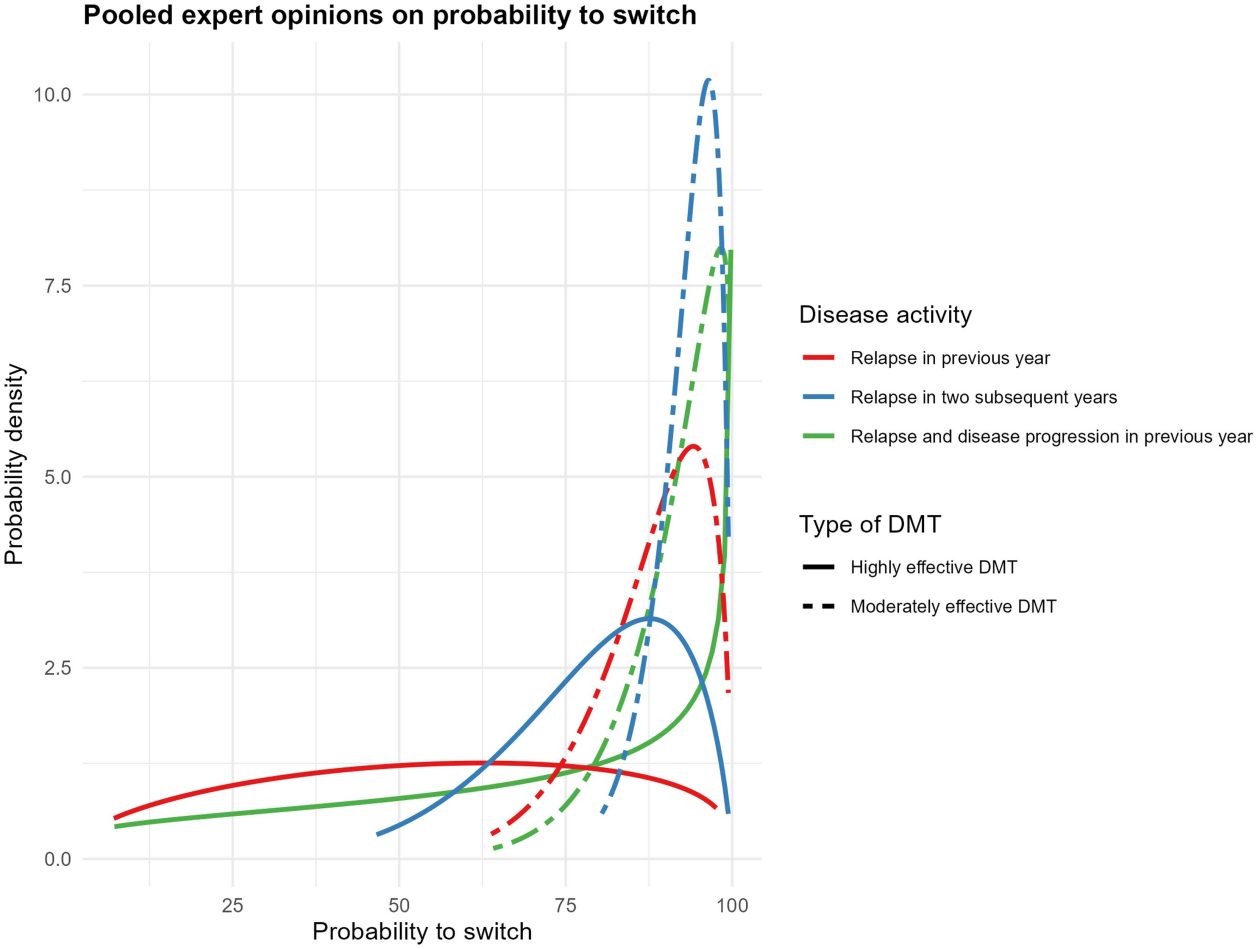
Probability density functions of pooled expert assessments of DMT switching probabilities due to disease activity. DMT, disease modyfing treatment.

The probability to switch DMT was lower for highly effective than for moderately effective DMTs. The probability to switch a patient when they had a relapse in the previous year was lower than in patients with relapses in two subsequent years and patients with a relapse and disability progression during the previous year (Figure 1). The narrower and higher peaks for moderately effective DMTs indicate greater expert consensus and certainty about switching probabilities, compared to the broader distributions for highly effective DMTs. The mean and standard deviation of the pooled answers are provided in Table 5. The probability density functions of the individual expert responses are available in Supplementary Figures 2–4. The mean minimum age the experts considered to stop DMT treatment in patient with stable disease was 62.1 years (SD 6.3 years) after a minimum mean duration of stable disease (i.e., no relapses or disability progression) of 14.0 years (SD 5.9 years) (Supplementary Figure 5).

**Table 5:** Pooled mean and standard deviations of the probabilities to switch DMT treatment due to disease activity.

Disease activity	Highly effective DMTs	Moderately effective DMTs
Relapse in previous year	53.8% ± 25.4%	87.3% ± 9.0%
Relapse in two subsequent years	78.4% ± 13.6%	93.0% ± 4.8%
Relapse and progression in previous year	66.3% ± 28.2%	90.9% ± 7.7%

DMT, disease modyfing treatment.

#### Leave-one-out analysis

The leave-one-out analysis indicated that pooled estimates were generally robust to the removal of a single expert (Table 6). For questions 3, 4, 5, 7, and 8, the maximum relative difference between the full-panel pooled mean and the maximum deviation in the leave-one-out analysis was less than 10% (ranging from 1.97 to 8.54%), suggesting strong agreement among the experts. For question 2, 6, and 9, the maximum relative difference was larger but remained below 25% (23.51, 21.38 and 16.09%, respectively). The expert responsible for the greatest deviation varied across these questions, suggesting natural heterogeneity in expert opinion rather than a systematic bias from one expert. Supplementary Figure 8 show the distribution of the outcomes of the full expert panel versus the leave-one-out analyses. When we use the maximum leave-one-out differences of the elicitation question as input in the model in a deterministic sensitivity analysis, the ranking of the sequences on net health benefit did not change, identifying RIT-CLA-PON-NAT is the most cost-effective treatment sequence.

**Table 6:** Results of leave-one-out analysis.

Question	Distribution	Pooled mean	Maximum absolute difference between leave-one-out analysis and pooled mean	Maximum relative difference between leave-one-out analysis and pooled mean	Most influential expert
Relapse in previous year on highly effective DMTs	Beta	0.5377	0.1264	23.51	1,111
Relapse in previous year on moderately effective DMTs	Beta	0.8728	0.0373	4.28	1,111
Relapse in two subsequent years on highly effective DMTs	Beta	0.7840	0.0669	8.54	1,111
Relapse in two subsequent years on moderately effective DMTs	Beta	0.9298	0.0183	1.97	1,111
Relapse and disease progression in previous year on highly effective DMTs	Beta	0.6633	0.1418	21.38	3,333
Relapse and disease progression in previous year on moderately effective DMTs	Beta	0.9090	0.0307	3.37	2,222
Minimum age considering stop DMT treatment when stable disease	Normal	62.1486	2.2507	3.62	2,222
Minimum duration of stable disease to consider stopping DMT treatment	Normal	13.9963	2.2526	16.09	4,444

DMT, disease modyfing treatment.

### Cost-effectiveness outcomes

**Figure 2:**
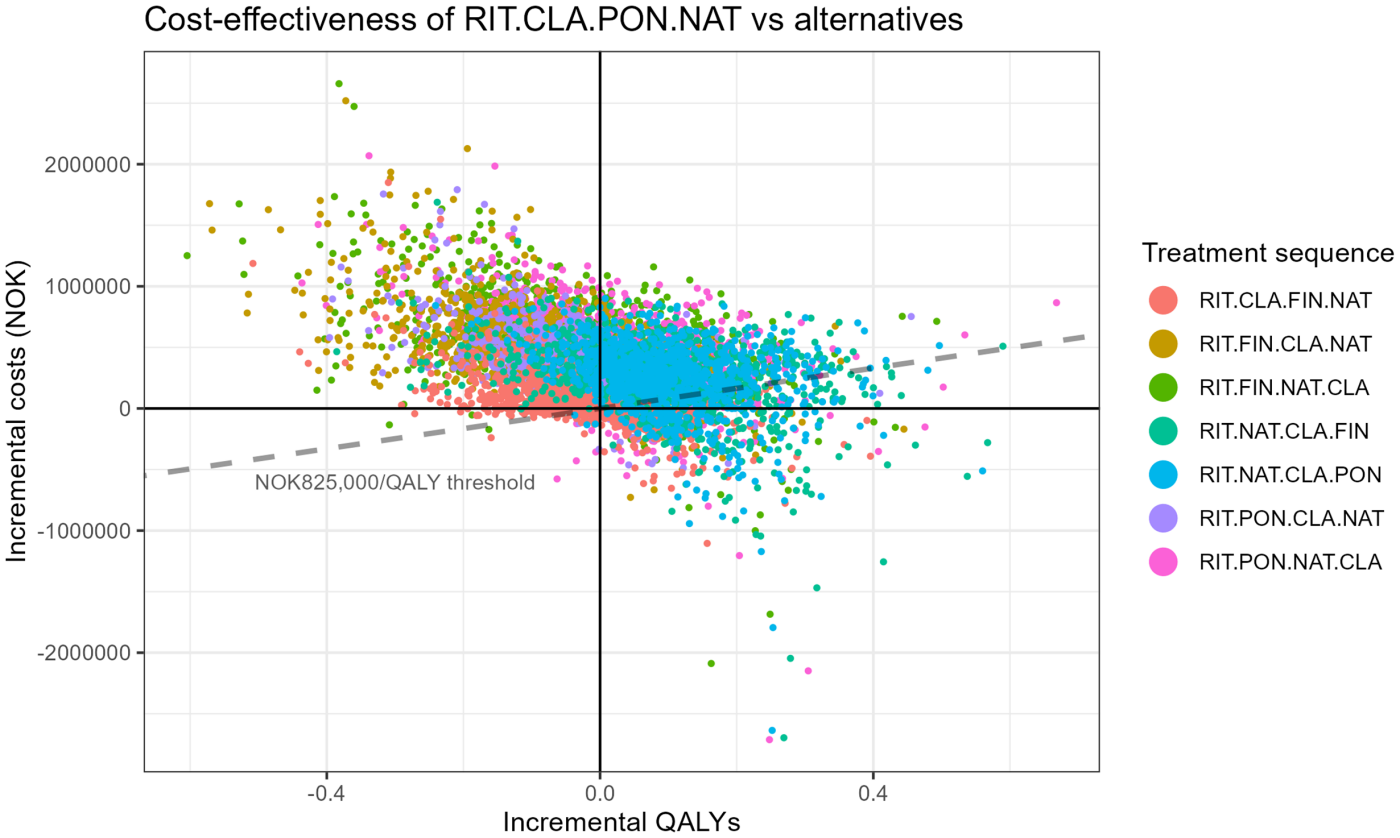
Cost-effectiveness plane of RIT-CLA-PON-NAT versus the 7 alternative treatment sequences starting with RIT based on probabilistic sensitivity analysis. RIT, rituximab; CLA, cladribine; PON, ponesimod; NAT, natalizumab.

Table 7 shows the fully incremental analysis of all sequences and Supplementary Figure 9 illustrates this in a cost-effectiveness frontier, both based on the mean outcomes of the probabilistic sensitivity analysis. The finding that the sequence CLA-PON-NAT was most cost-effective in MS patients who started with rituximab was considerably certain as shown in the cost-effectiveness plane in Figure 2. Figure 2 shows the uncertainty in the outcomes based on the probabilistic sensitivity analysis in which all parameters are varied across their distribution. The cost-effectiveness acceptability curve in Supplementary Figure 6 shows that it is 66.1%–96.4% certain that this sequence is cost-effective compared to the other possible sequences starting with rituximab. It is highly certain (96.4%) that RIT-CLA-PON-NAT is cost-effective compared to RIT-FIN-CLA-NAT. There is more overlap between RIT-CLA-PON-NAT and that RIT-CLA-FIN-NAT, but it is still 66.1% certain that RIT-CLA-PON-NAT is cost-effective. The probability that second line CLA is cost-effective (either followed by PON or FIN) is more than 75%.

**Table 7:** Fully incremental analysis based on probabilistic sensitivity analysis.

Treatment sequence	Total costs	Total QALYs	Incremental costs	Incremental QALYs	ICER	NHB	Status
RIT-CLA-PON-NAT	13,648,618	11.20	NA	NA	NA	−5.34	ND
RIT-CLA-FIN-NAT	13,701,778	11.17	NA	NA	NA	−5.44	D
RIT-NAT-CLA-PON	13,891,928	11.30	243,309	0.101	2,397,355	−5.54	ND
RIT-NAT-CLA-FIN	13,914,246	11.29	NA	NA	NA	−5.58	D
RIT-PON-CLA-NAT	14,003,800	11.17	NA	NA	NA	−5.80	D
RIT-PON-NAT-CLA	14,083,130	11.23	NA	NA	NA	−5.84	D
RIT-FIN-CLA-NAT	14,162,288	11.12	NA	NA	NA	−6.05	D
RIT-FIN-NAT-CLA	14,208,101	11.17	NA	NA	NA	−6.06	D

RIT, rituximab; CLA, cladribine tablets; PON, ponesimod; NAT, natalizumab; FIN, fingolimod; QALY, quality adjusted life year; ICER, incremental cost-effectiveness ratio. D, dominated; ND, not dominated; NHB, net health benefit at willingness to pay of 825.000 NOK.

## Discussion

This study estimated the cost-effectiveness of treatment pathways starting with rituximab for the treatment of relapsing remitting MS in Norway. To do so, we needed to parameterize the switch behavior of neurologists, and we did so with using structured expert elicitation. Neurologists are more likely to switch patients to a new therapy when the patient is on a moderately effective DMT and their views were relatively homogeneous in this respect. The cost-effectiveness model, including these switch probabilities, demonstrated that the sequence rituximab-cladribine tablets-ponesimod-natalizumab is most cost-effective and that the sequence rituximab-natalizumab-cladribine tablets-ponesimod yields more QALYs (0.101 QALY), but at additional costs (243,309 NOK), causing the incremental cost-effectiveness ratio to lie above the Norwegian threshold.

Heterogeneity regarding treatment *outcomes* is relatively well-studied (26). Heterogeneity in *responses* of clinicians to treatment outcomes has received less attention. As demonstrated here, neurologists may have differing responses to disease activity in patients and may not commence a subsequent treatment when presented with similar patient outcomes. This heterogeneity was most pronounced for what to do when there is disease activity on highly effective DMTs. This is not surprising: most first line rituximab initiations have occurred during the last 10 years in Norway, and according to our model, the mean time on treatment is 15.2 years. As such, no consensus on optimal clinical practice may have emerged in Norway on what to do with patients who have disease activity or adverse effects including hypogammaglobulinemia on rituximab. Regarding the divergent views among interviewed neurologists on the age at which DMTs can be stopped as well as the required period of non-active disease; this may well reflect the current scientific state of knowledge, or lack thereof, regarding the consequences of discontinuing treatments at different ages.

This is the first study of its kind for Norway, comparing the cost-effectiveness of sequences rather than two individual treatments. There is also limited information available from “traditional” (non-sequence) cost-effectiveness studies on MS in Norway. The Norwegian Institute of Public Health performed a cost-effectiveness analysis of DMTs for RRMS in 2019 using a Markov model. In the public report, the cost-effectiveness results were redacted and the comparisons between DMTs were different than in our study limiting meaningful comparisons of results. The report suggested that cladribine tablets are both less effective and more costly than natalizumab and fingolimod (ponesimod was not included). These results are in line with our findings for natalizumab, but for fingolimod we found that cladribine tablets were more effective, which is similar to what has been found in network meta-analyses (6, 27, 28), and real-world studies (29, 30).

There are several limitations to this study. First, the results of this study only apply to Norway and its specific treatment paradigm, and DMT acquisition costs. Second, we included only four clinical experts in the structured expert elicitation. The Norwegian guideline for cost-effectiveness studies does not specify an optimal number of experts to interview, but four would probably be considered a minimum. The four included experts were, as is ideal, associated with different treatment centers. Third, our study included list prices rather than negotiated prices. While this is in line with the Norwegian guideline, which requests the use of maximum retail prices without VAT (13), discounts that are centrally negotiated may impact expenditures from a societal perspective and change outcomes. Fourth, autologous hematopoietic stem cell transplantation (aHSCT) was not included in this model. At the time of expert interviews aHSCT was offered in Norway through the RAM-MS study (NCT03477500), but it has later been officially accepted as a treatment option for patients who have disease activity on highly effective DMTs. Costs of aHSCT have been well studied in Norway and an observational study suggests good long-term efficacy (31, 32). Evidence from other countries support that aHSCT may induce durable remission (33), possibly superior to natalizumab and similar to ocrelizumab (34), but controlled trials, such as StarMS (NCT03477500) and RAM-MS (NCT03477500) (35), are still ongoing. The limited evidence on the risk–benefit profile of aHSCT after rituximab caused us to refrain from including it as a treatment option (36). Future model updates, when more information is available, should include aHSCT to understand how its costs and benefits compare with other treatment modalities. Fifth, some assumptions were required for rituximab regarding disease progression and discontinuation. While these assumptions were confirmed by clinical experts, and the discontinuation relative risk was sourced from published Norwegian registry data (3), direct comparison data would be more reliable. These assumptions only have a very limited impact on incremental outcomes as rituximab is fixed in line 1 for all compared treatment sequences. As such, different assumptions would similarly affect the total costs and effects for all sequences. Sixth, specific rebound risk after fingolimod and natalizumab has not been included in the model. It has been described that patients switching from fingolimod have a higher rebound risk when switched to cladribine tablets than when switched to rituximab (37). Also for natalizumab, swift initiation of any follow-up therapy with a fast onset of action is required to avoid rebounds (38). Recent evidence showed that sustained suppression of disease activity can be achieved when switching from natalizumab to cladribine tablets (39–41). Despite this, if the cause for switch is managing progressive multifocal leukoencephalopathy (PML) risk, extending the interval of natalizumab dosing might be a more appropriate management strategy than initiating another DMT. The exclusion of the rebound risk in our study is unlikely to impact the main findings: including rebound risks would most likely result in sequences with natalizumab or fingolimod in line 2 yielding less QALYs further supporting the current findings. Seventh, due to the absence of Norwegian-specific data, the costs of PML were derived from a Dutch estimate and converted to Norwegian Krone and the disutility of PML was sourced from the Dutch reimbursement dossier for natalizumab, where it was based on expert opinion. While this may introduce some uncertainty, the low incidence of PML makes it unlikely to have influenced the conclusions of our analyses. Finally, in clinical practice, CD20 and CD19 B-cell counts define when a next DMT can be initiated following loss of response. Our model does not explicitly model the level of B-cell repopulation thresholds, but works on a higher level of abstraction. However, in our model, treatment switches occur with probabilities derived from Norwegian neurologists which also reflects the assessment of repopulation thresholds by neurologists.

## Conclusion

This study showed that neurologists’ views on when to switch patients are heterogeneous when patients experience disease progression and are relatively homogeneous when patients experience relapse. For both types of disease activity neurologists are more likely to switch patients to a new therapy when the patient is on a moderately effective DMT compared to a highly effective DMT. Patients receiving first line rituximab may experience new disease activity or side-effects that necessitate a change in treatment. If a switch from rituximab to another DMT is the preferred clinical course of action, this study suggests that it is most cost-effective to switch to cladribine tablets followed by ponesimod and natalizumab.

## Data Availability

The original contributions presented in the study are included in the article/Supplementary material, further inquiries can be directed to the corresponding author.

## References

[1] Norwegian Directorate of Health (Helsedirektoratet). Multippel sklerose. Helsedirektoratet (2025). Available online at: https://www.helsedirektoratet.no/retningslinjer/multippel-sklerose (Accessed July 3, 2025)

[2] Svenningsson A Frisell T Burman J Salzer J Fink K Hallberg S . Safety and efficacy of rituximab versus dimethyl fumarate in patients with relapsing-remitting multiple sclerosis or clinically isolated syndrome in Sweden: a rater-blinded, phase 3, randomised controlled trial. Lancet Neurol. (2022) 21:693–703. doi: 10.1016/S1474-4422(22)00209-5, 35841908

[3] Rød BE Høgestøl EA Torkildsen Ø Bjørnevik K Gran JM Øverås MH . Comparative effectiveness of rituximab and cladribine in relapsing–remitting multiple sclerosis: a target trial emulation. Mult Scler. (2025) 31:13524585251342727. doi: 10.1177/13524585251342727, 40415655 PMC12228892

[4] Huygens S Versteegh M. Modeling the cost-utility of treatment sequences for multiple sclerosis. Value Health. (2021) 24:1612–9. doi: 10.1016/j.jval.2021.05.020, 34711361

[5] Versteegh MM Huygens SA Wokke BWH Smolders J. Effectiveness and cost-effectiveness of 360 disease-modifying treatment escalation sequences in multiple sclerosis. Value Health. (2022) 25:984–91. doi: 10.1016/j.jval.2021.11.1363 35667786

[6] Corsten CEA Huygens SA Versteegh MM Wokke BHA Smets I Smolders J. Benefits of sphingosine-1-phosphate receptor modulators in relapsing MS estimated with a treatment sequence model. Mult Scler Relat Disord. (2023) 80:105100. doi: 10.1016/j.msard.2023.105100, 37944195

[7] Versteegh MM Huygens SA. Exit strategies in patients with stable MS: cost-effectiveness of extended interval dosing of ocrelizumab and natalizumab versus de-escalating to cladribine. Mult Scler Relat Disord. (2025) 102:106625. doi: 10.1016/j.msard.2025.106625, 40714725

[8] Jankovic D Horscroft J Lee D Bojke L Soares M. STEER: open access resources for conducting structured expert elicitation for health care decision making. Med Decis Mak. (2025) 45:627–39. doi: 10.1177/0272989X251343013, 40567045

[9] Jankovic D Soares M Bojke L Horscroft J Lee D. R Code for Building Bespoke Shiny Apps for Conducting SEE. London: STEER (2022).

[10] Horscroft J Lee D Jankovic D Soares M Bojke L. Structured expert elicitation for healthcare decision making: a practical guide (2025). Available online at: https://www.york.ac.uk/media/che/documents/Structured%20expert%20elicitation%20for%20healthcare%20decision%20making%20A%20practical%20guide.pdf (Accessed October 06, 2025).

[11] Smets I Versteegh M Huygens S Corsten C Wokke B Smolders J. Health-economic benefits of anti-CD20 treatments in relapsing multiple sclerosis estimated using a treatment-sequence model. Mult Scler J Exp Transl Clin. (2023) 9:20552173231189398. doi: 10.1177/20552173231189398, 37529628 PMC10387699

[12] Smets I Versteegh M Huygens S Wokke B Smolders J. Benefits of early highly effective versus escalation treatment strategies in relapsing multiple sclerosis estimated using a treatment-sequence model. Mult Scler. (2024) 30:1016–25. doi: 10.1177/13524585241258692, 38859625 PMC11290018

[13] Norwegian Medical Products Agency. Submission Guidelines for Single Technology Assessment of Medicinal Products. Oslo: Norwegian Medical Products Agency (2024).

[14] Campbell JA Henson GJ Ngwa VF Ahmad H Taylor BV van der Mei I . Estimation of transition probabilities from a large cohort (> 6000) of Australians living with multiple sclerosis (MS) for changing disability severity classifications, MS phenotype, and disease-modifying therapy classifications. PharmacoEconomics. (2024) 43:223–39. doi: 10.1007/s40273-024-01417-4, 39095665 PMC11782298

[15] Liu Z Liao Q Wen H Zhang Y. Disease modifying therapies in relapsing-remitting multiple sclerosis: a systematic review and network meta-analysis. Autoimmun Rev. (2021) 20:102826. doi: 10.1016/j.autrev.2021.102826, 33878488

[16] Hauser SL Waubant E Arnold DL Vollmer T Antel J Fox RJ . B-cell depletion with rituximab in relapsing-remitting multiple sclerosis. N Engl J Med. (2008) 358:676–88. doi: 10.1056/NEJMoa0706383, 18272891

[17] Ciron J Bourre B Castelnovo G Guennoc AM de Sèze J Ben-Amor AF . Holistic, long-term management of people with relapsing multiple sclerosis with cladribine tablets: expert opinion from France. Neurol Ther. (2024) 13:503–18. doi: 10.1007/s40120-024-00589-7, 38488979 PMC11136930

[18] Zorginstituut Nederland. Farmacotherapeutisch Rapport Natalizumab (Tysabri®) Bij ‘zeer Actieve Relapsing Remitting Multiple Sclerose (RRMS) Met Een Hoge Ziekteactiviteit Ondanks Behandeling Met Interferon Bèta. Diemen: Zorginstituut Nederland (2014).

[19] Norwegian Institute of Public Health. Disease modifying treatments for relapsing remitting multiple sclerosis (2019). Available online at: https://www.fhi.no/en/publ/2019/Disease-modifying-treatments-for-relapsing-remitting-multiple-sclerosis/ (Accessed August 27, 2019)

[20] Svendsen B Myhr K-M Nyland H Aarseth JH. The cost of multiple sclerosis in Norway. Eur J Health Econ. (2012) 13:81–91. doi: 10.1007/s10198-010-0286-7, 21080024 PMC3249563

[21] Kobelt G Berg J Atherly D Hadjimichael O. Costs and quality of life in multiple sclerosis: a cross-sectional study in the United States. Neurology. (2006) 66:1696–702. doi: 10.1212/01.wnl.0000218309.01322.5c, 16769943

[22] Uitdehaag B Kobelt G Berg J Capsa D Dalén J The European Multiple Sclerosis Platform. New insights into the burden and costs of multiple sclerosis in Europe: results for the Netherlands. Mult Scler. (2017) 23:117–29. doi: 10.1177/1352458517708663, 28643595

[23] Orme M Kerrigan J Tyas D Russell N Nixon R. The effect of disease, functional status, and relapses on the utility of people with multiple sclerosis in the UK. Value Health. (2007) 10:54–60. doi: 10.1111/j.1524-4733.2006.00144.x, 17261116

[24] Pokorski RJ. Long-term survival experience of patients with multiple sclerosis. J Insur Med. (1997) 29:101–6. 10169627

[25] Zimmermann M Brouwer E Tice JA Seidner M Loos AM Liu S . Disease-modifying therapies for relapsing-remitting and primary progressive multiple sclerosis: a cost-utility analysis. CNS Drugs. (2018) 32:1145–57. doi: 10.1007/s40263-018-0566-9, 30141001

[26] Sormani MP Chataway J Kent DM Marrie RA. Assessing heterogeneity of treatment effect in multiple sclerosis trials. Mult Scler. (2023) 29:1158–61. doi: 10.1177/13524585231189673, 37555493 PMC10413777

[27] Siddiqui MK Khurana IS Budhia S Hettle R Harty G Wong SL. Systematic literature review and network meta-analysis of cladribine tablets versus alternative disease-modifying treatments for relapsing–remitting multiple sclerosis. Curr Med Res Opin. (2018) 34:1361–71. doi: 10.1080/03007995.2017.1407303, 29149804

[28] McCool R Wilson K Arber M Fleetwood K Toupin S Thom H . Systematic review and network meta-analysis comparing ocrelizumab with other treatments for relapsing multiple sclerosis. Mult Scler Relat Disord. (2019) 29:55–61. doi: 10.1016/j.msard.2018.12.040, 30677733

[29] Spelman T Ozakbas S Alroughani R Terzi M Hodgkinson S Laureys G . Comparative effectiveness of cladribine tablets versus other oral disease-modifying treatments for multiple sclerosis: results from MSBase registry. Mult Scler. (2023) 29:221–35. doi: 10.1177/13524585221137502, 36433775 PMC9925904

[30] Signori A Saccà F Lanzillo R Maniscalco GT Signoriello E Repice AM . Cladribine vs other drugs in MS. Neurology Neuroimmunol Neuroinflamm. (2020) 7:e878. doi: 10.1212/NXI.0000000000000878, 32801167 PMC7641098

[31] Gottschlich KN Zolic-Karlsson Z Aas E Kvistad SAS Bø L Torkildsen Ø . Healthcare utilization and costs associated with autologous haematopoietic stem cell transplantation in Norwegian patients with relapsing remitting multiple sclerosis. Mult Scler Relat Disord. (2024) 84:105507. doi: 10.1016/j.msard.2024.105507, 38412758

[32] Kvistad CE Lehmann AK Kvistad SAS Holmøy T Lorentzen ÅR Trovik LH . Autologous hematopoietic stem cell transplantation for multiple sclerosis: long-term follow-up data from Norway. Mult Scler. (2024) 30:751–4. doi: 10.1177/13524585241231665, 38345003 PMC11071593

[33] Boffa G Massacesi L Inglese M Mariottini A Capobianco M Moiola L . Long-term clinical outcomes of hematopoietic stem cell transplantation in multiple sclerosis. Neurology. (2021) 96:e1215–26. doi: 10.1212/WNL.0000000000011461, 33472915

[34] Kalincik T Sharmin S Roos I Freedman MS Atkins H Burman J . Comparative effectiveness of autologous hematopoietic stem cell transplant vs fingolimod, natalizumab, and ocrelizumab in highly active relapsing-remitting multiple sclerosis. JAMA Neurol. (2023) 80:702–13. doi: 10.1001/jamaneurol.2023.1184 37437240 PMC10186210

[35] Brittain G Petrie J Duffy KEM Glover R Hullock K Papaioannou D . Efficacy and safety of autologous haematopoietic stem cell transplantation versus alemtuzumab, ocrelizumab, ofatumumab or cladribine in relapsing remitting multiple sclerosis (StarMS): protocol for a randomised controlled trial. BMJ Open. (2024) 14:e083582. doi: 10.1136/bmjopen-2023-083582, 38316583 PMC10860024

[36] Noui Y Zjukovskaja C Silfverberg T Ljungman P Kultima K Tolf A . Factors associated with outcomes following autologous haematopoietic stem cell transplantation for multiple sclerosis. J Neurol Neurosurg Psychiatry. (2025) 96:966–74. doi: 10.1136/jnnp-2024-335512, 40050008 PMC12505080

[37] Nygaard GO Torgauten H Skattebøl L Høgestøl EA Sowa P Myhr K-M . Risk of fingolimod rebound after switching to cladribine or rituximab in multiple sclerosis. Mult Scler Relat Disord. (2022) 62:103812. doi: 10.1016/j.msard.2022.103812, 35462167

[38] González-Suarez I de Rodríguez Antonio L Orviz A Moreno‐García S Valle‐Arcos MD Matias‐Guiu JA . Catastrophic outcome of patients with a rebound after natalizumab treatment discontinuation. Brain Behav. (2017) 7:e00671. doi: 10.1002/brb3.671, 28413713 PMC5390845

[39] Sguigna PV Hussain RZ Okai A Blackburn KM Tardo L Madinawala M . Cladribine tablets after treatment with natalizumab (CLADRINA) – rationale and design. Ther Adv Neurol Disord. (2024) 17:17562864241233858. doi: 10.1177/17562864241233858, 38585373 PMC10996356

[40] Sguigna P. Two-year findings on the safety and efficacy of cladribine tablets after treatment with natalizumab (CLADRINA trial). In: Proceedings of the 2025 ACTRIMS Forum. Madison, WI: ACTRIMS (2025)

[41] Vlahovic L Gervasi-Follmar T Stuchiner T Chen C Rutledge D Avila R . Single-center retrospective safety analysis of people with multiple sclerosis treated with higher efficacy disease modifying therapies. Mult Scler. (2025) 31:1391–471. doi: 10.1177/1352458525137053340980836

