# Supplementary material for: Cost-effectiveness of treatment sequences following first-line rituximab in relapsing-remitting multiple sclerosis: a Norwegian microsimulation study
Source: Front Neurol. 2026 Mar 18;17:1783506. doi: 10.3389/fneur.2026.1783506 (PMC13038511; doi:10.3389/fneur.2026.1783506)
Supplement: Supplementary file 1 [file Data_Sheet_1.docx]

**Supplemental material:**

Expert elicitation questionnaire on MS treatment sequences in clinical practice in Norway

*Answers used as input for a microsimulation model on cost-effectiveness of treatment sequences in relapse remitting multiple sclerosis patients.*

Norwegian setting

- Q1: Given the DMTs that are currently available for the treatment of MS, how many DMTs (lines of treatment) will a patient typically undergo over their lifetime?
- Q2: Is it correct to assume that newly-diagnosed RRMS patients in Norway (without contraindications for highly-effective DMTs) generally receive **rituximab** as a first-line treatment?
- Q3: Are we correct to assume that there is very limited use of non-highly effective DMTs (dimethyl fumarate, glatiramer acetate, teriflunomide and interferons) in Norway?
  - If yes, should they be excluded from the model?
  - Are they generally prescribed after failure on rituximab?

Switching rules

*Answers elicited with structured expert elicitation tool STEER*

- Q4a: What proportion of patients with a **relapse in the previous year** while on a **highly effective DMT** will switch to another DMT?
- Q4b: What proportion of patients with a **relapse in the previous year** while on a **moderately effective** **DMT** will switch to another DMT?
- Q5a: What proportion of patients with a **relapse in two subsequent years** while on a **highly effective DMT** will switch to another DMT?
- Q5b: What proportion of patients with a **relapse in two subsequent years** while on a **moderately effective DMT** will switch to another DMT?
- Q6a: What proportion of patients with a **relapse and disease progression in the previous year** while on a **highly effective DMT** will switch to another DMT?
- Q6b: What proportion of patients with a **relapse and disease progression in the previous year** while on a **moderately effective** **DMT** will switch to another DMT?

Moderately effective DMTs: interferons, glatiramer, fumarates, and teriflunomide
Highly effective DMTs: alemtuzumab, cladribine, natalizumab, ocrelizumab, ofatumumab, fingolimod, ozanimod, ponesimod

Selection of next DMT

- Q7: When you decide to switch DMTs due to lack of efficacy, would you generally switch the patient to a DMT within the same class?
- Q8: Would you also consider switching a patient from **high to moderate efficacy** DMTs (moderate = interferons, glatiramer, fumarates and teriflunomide)?
- Q9: Would you consider switching a patient **from oral to injectable** DMTs?
- Q10: Re-treatment with cladribine
  - a: Under which conditions would you re-initiate cladribine treatment (i.e. prescribe a third cycle of treatment)?
  - b: How many years after the first dosage of cladribine would you prescribe this re-treatment?
  - c: How many dosages would you prescribe? 1 or 2?
- Q11: Should we include alemtuzumab as a **last resort treatment** or should we **exclude** it from the model? (continue with 12 if yes, otherwise skip to 13)
- Q12: Re-treatment with alemtuzumab
  - a: Under which conditions would you re-initiate alemtuzumab treatment (i.e. prescribe a third cycle of treatment)?
  - b: How many years after the first dosage of alemtuzumab would you prescribe this re-treatment?

Stopping DMT treatment

*Answers elicited with structured expert elicitation tool STEER*

- Q13: Stopping DMT treatment
  - a: Would you consider stopping a DMT when a patient has stable disease (i.e. no disability progression and relapses)? (continue if yes)
  - b: What is the **age** from which you would consider stopping treatment under stable disease?
  - c: What is the **number of years** a patients should have **stable disease** for you to consider stopping treatment?

Representativeness

- Q14: Do you feel that the way you treat MS patients is representative of how MS patients are treated in the Norway?

# Supplemental figures


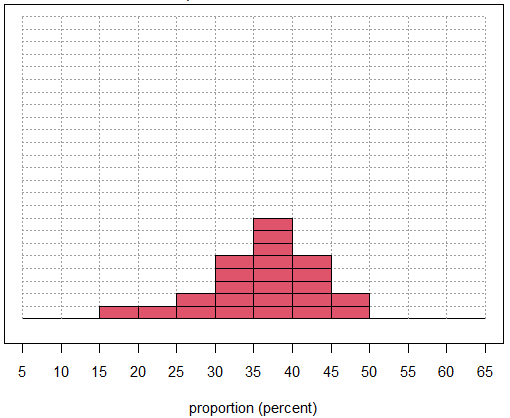


Supplemental figure 1. Example of ‘chips and bins’ method for structured expert elicitation.


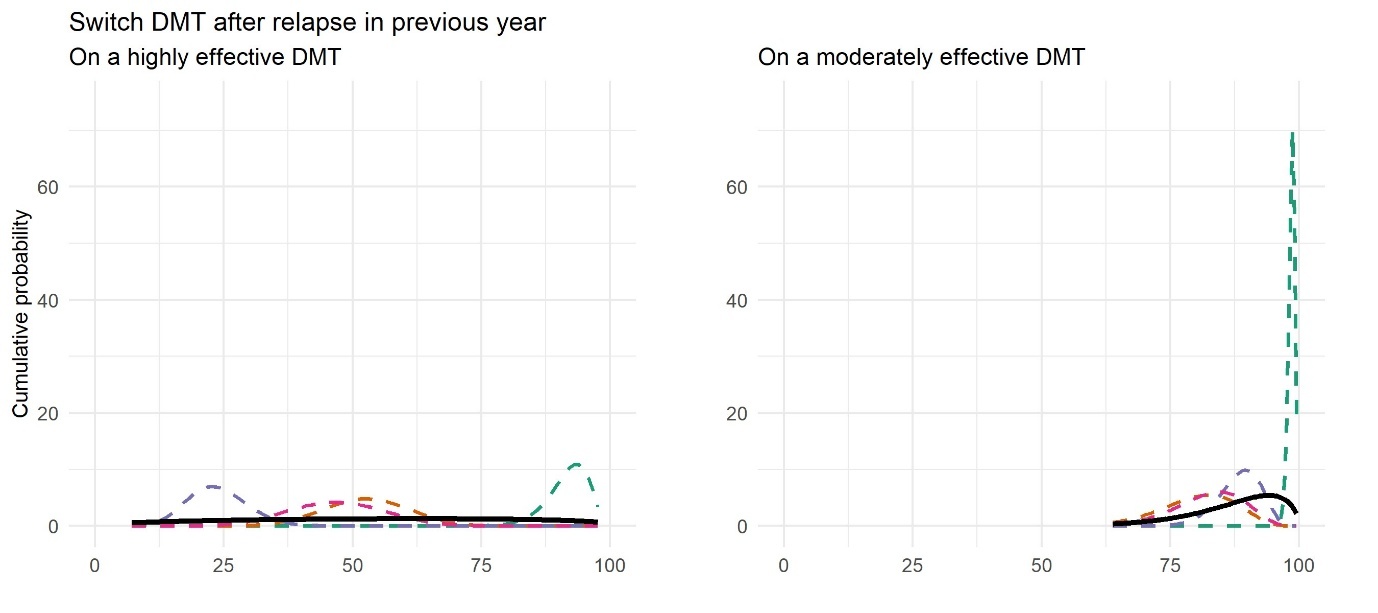


Supplemental figure 2. Expert assessments of DMT switching probabilities after a relapse in the previous year. Individual expert responses (dashed) and pooled estimates (solid) by disease activity scenario and while on highly or moderately effective DMTs.


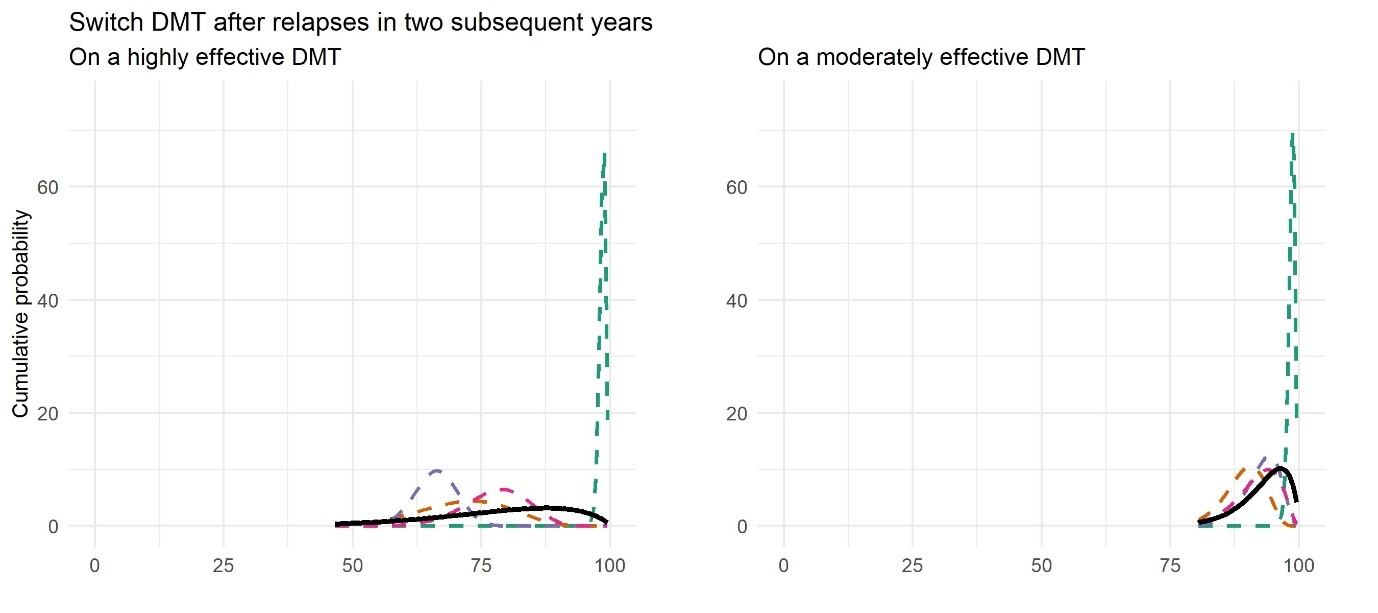


Supplemental figure 3. Expert assessments of DMT switching probabilities after a relapse in two subsequent years. Individual expert responses (dashed) and pooled estimates (solid) by disease activity scenario and while on highly or moderately effective DMTs.


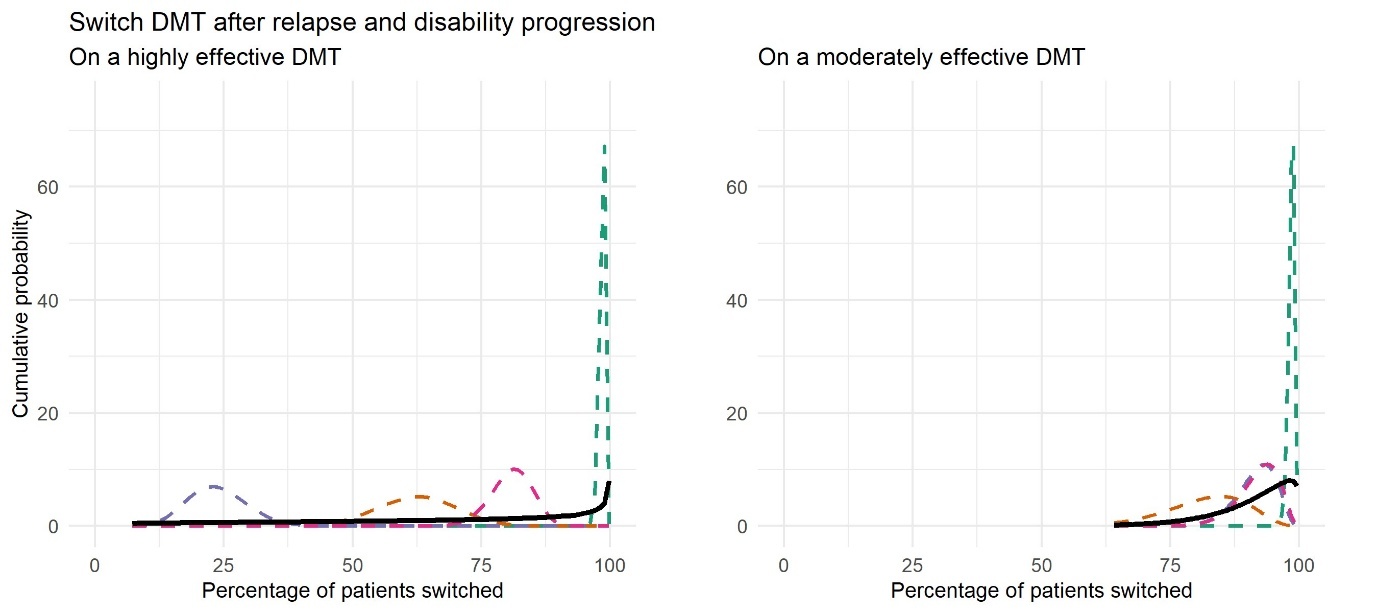


Supplemental figure 4. Expert assessments of DMT switching probabilities after a relapse and disability progression in the previous year. Individual expert responses (dashed) and pooled estimates (solid) by disease activity scenario and while on highly or moderately effective DMTs.


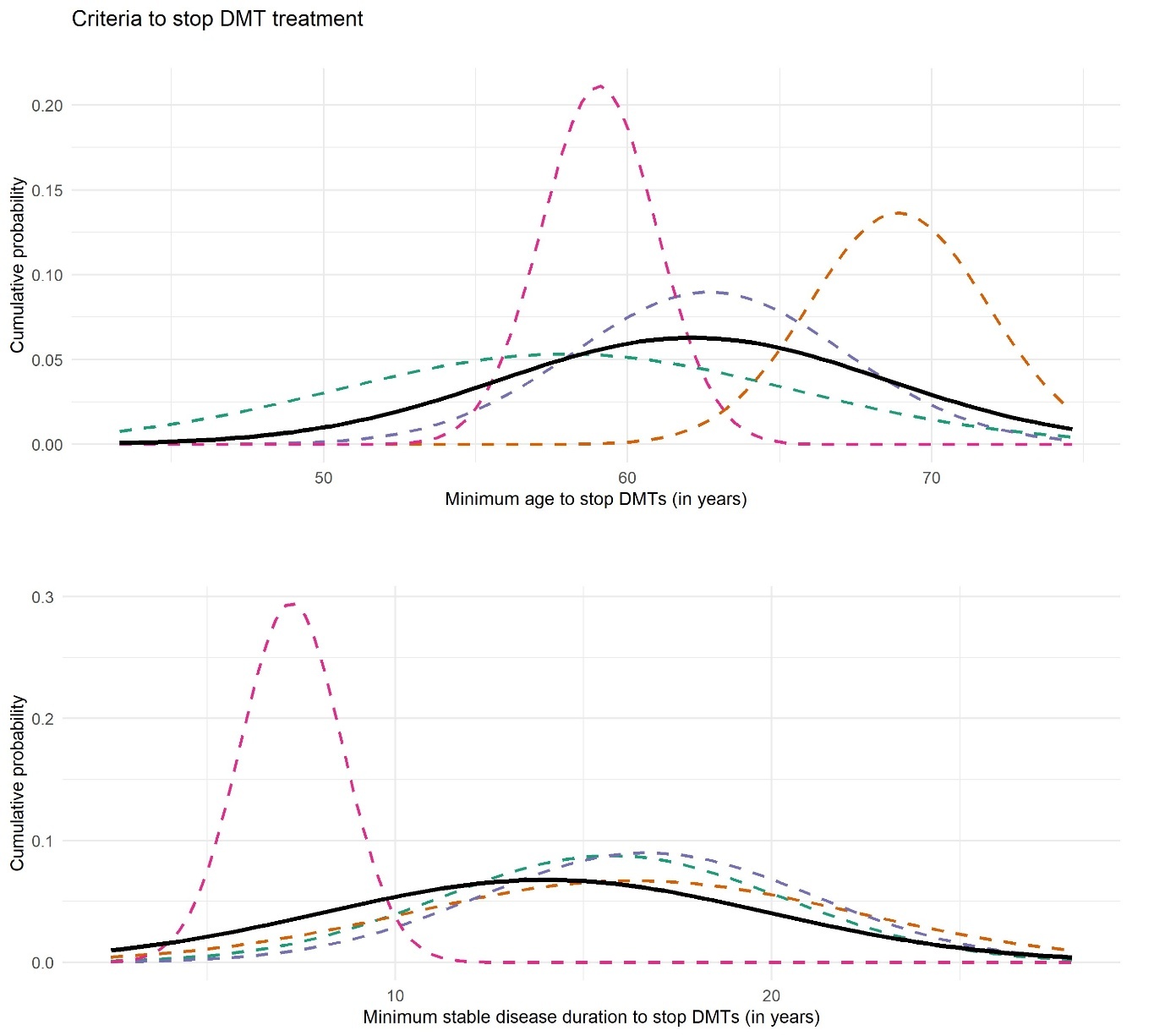


Supplemental figure 5. Expert assessments of minimum age and stable disease duration to stop DMTs. Individual expert responses (dashed) and pooled estimates (solid) by disease activity scenario and while on highly or moderately effective DMTs.


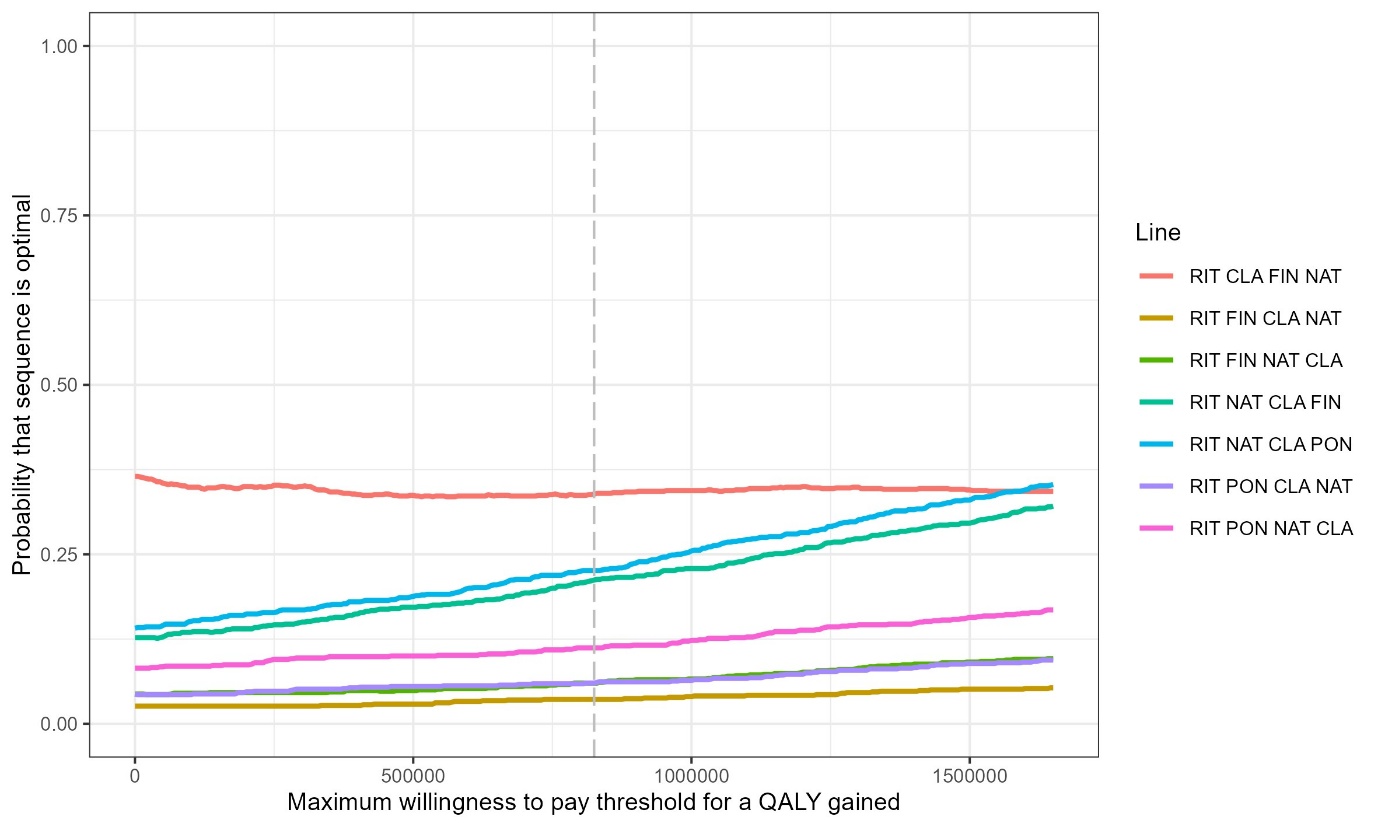


Supplemental figure 6. Cost-effectiveness acceptability curve of RIT-CLA-PON-NAT versus the 7 alternative treatment sequences starting with RIT.

| **Question 2: Relapse in previous year on highly effective DMTs** | |
| --- | --- |
| Expert 1111  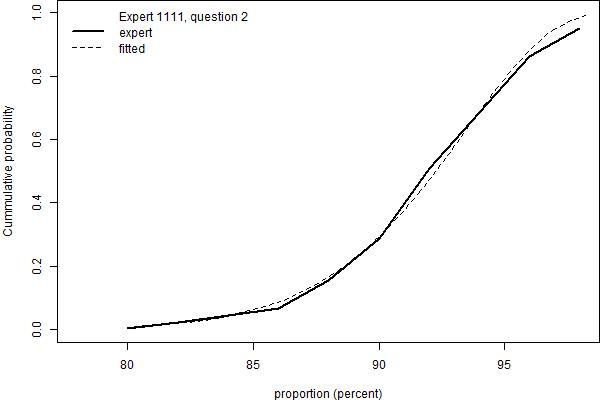 | Expert 2222  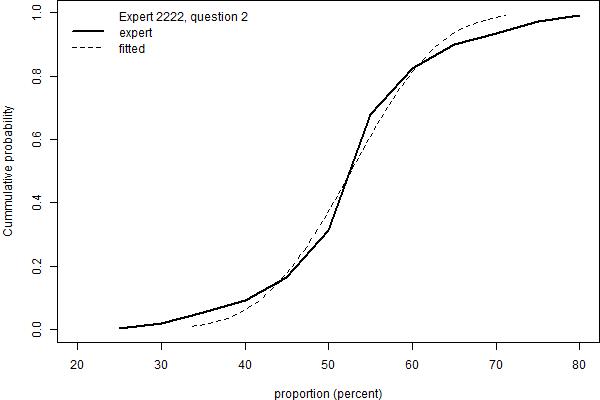 |
| Expert 3333  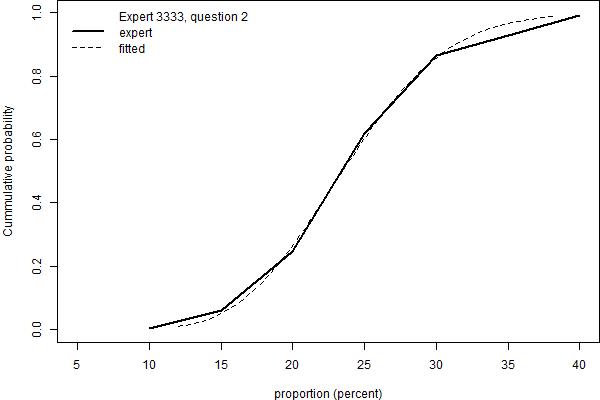 | Expert 4444  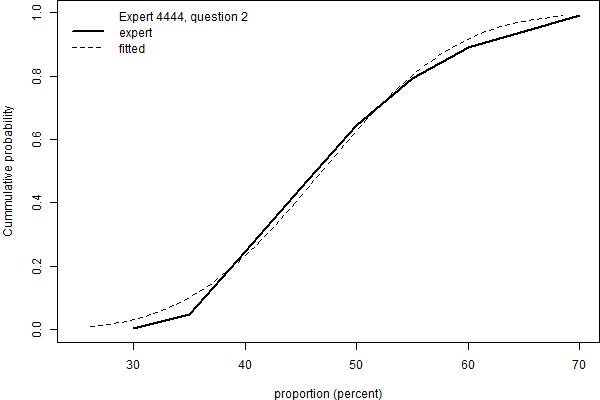 |
| **Question 3: Relapse in previous year on moderately effective DMTs** | |
| Expert 1111  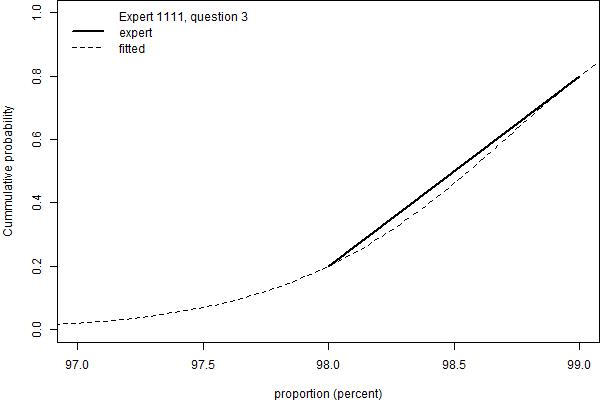 | Expert 2222  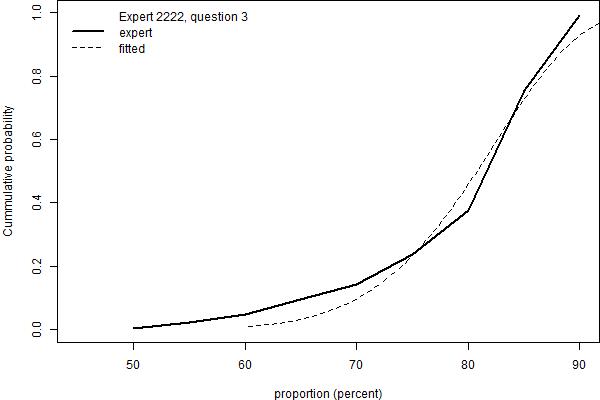 |
| Expert 3333  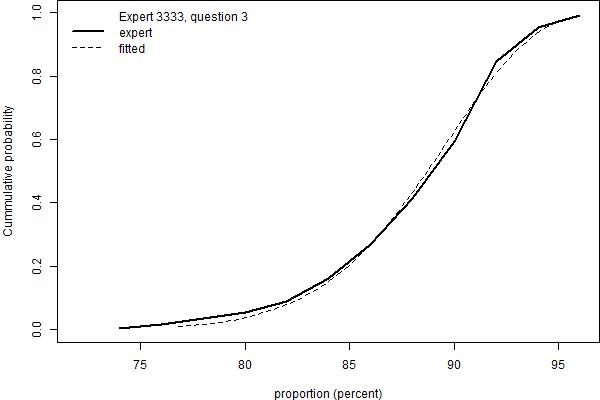 | Expert 4444  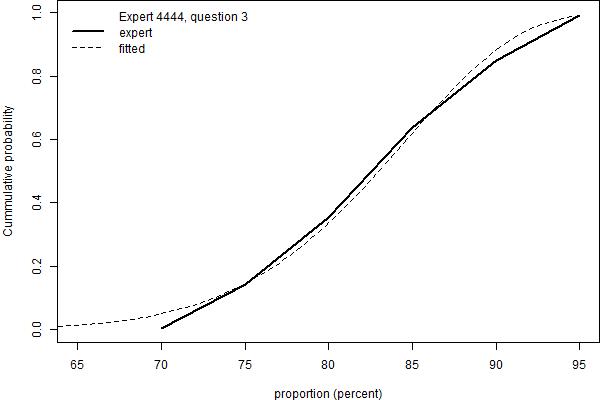 |
| **Question 4: Relapse in two subsequent years on highly effective DMTs** | |
| Expert 1111  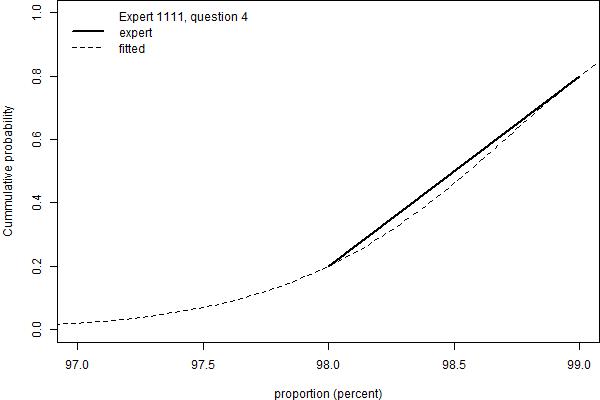 | Expert 2222  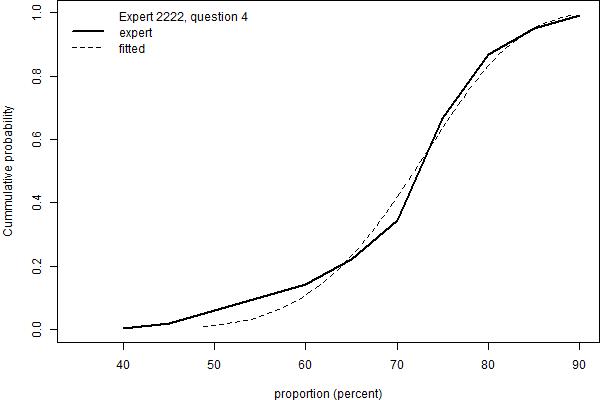 |
| Expert 3333  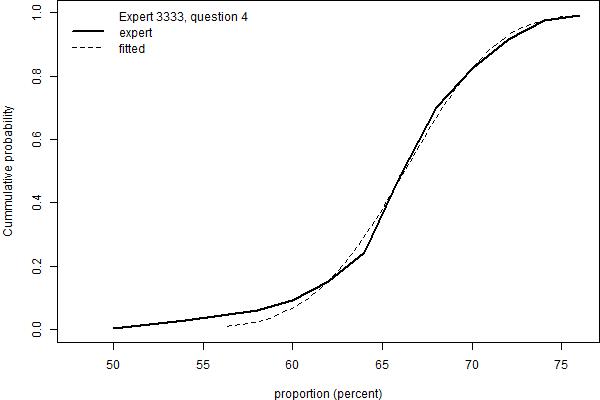 | Expert 4444  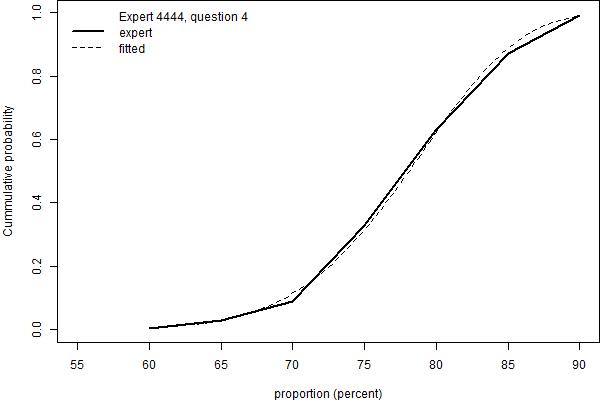 |
| **Question 5: Relapse in two subsequent years on moderately effective DMTs** | |
| Expert 1111  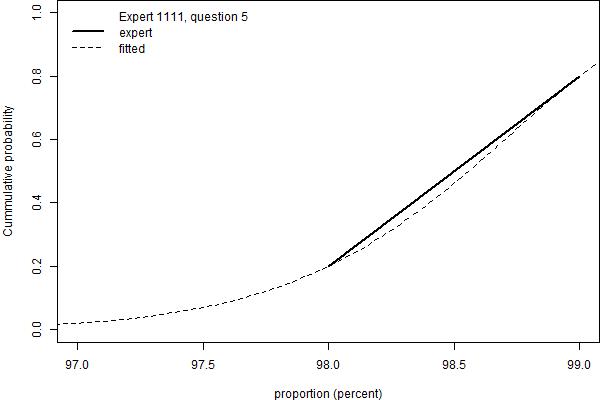 | Expert 2222  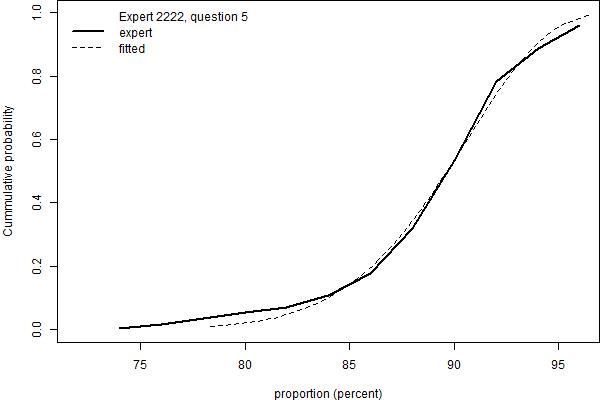 |
| Expert 3333  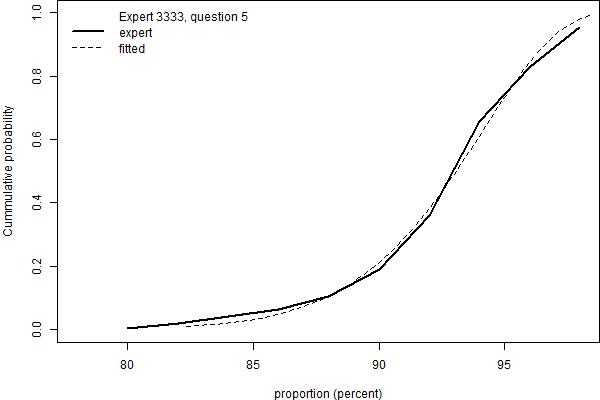 | Expert 4444  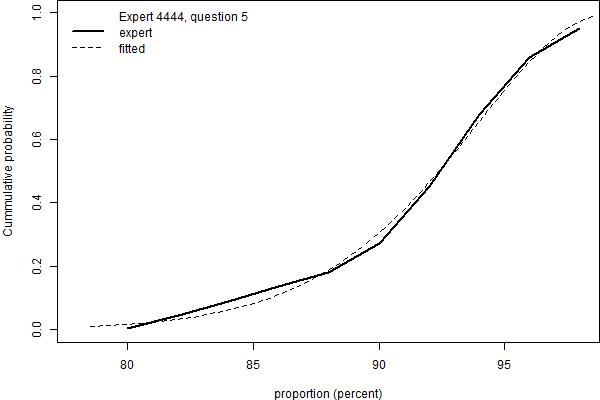 |
| **Question 6: Relapse and disease progression in previous year on highly effective DMTs** | |
| Expert 1111  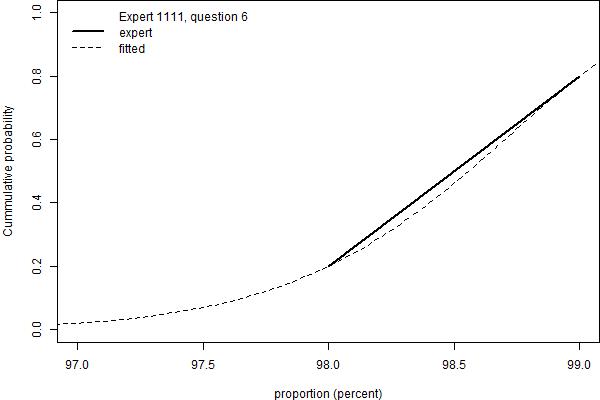 | Expert 2222  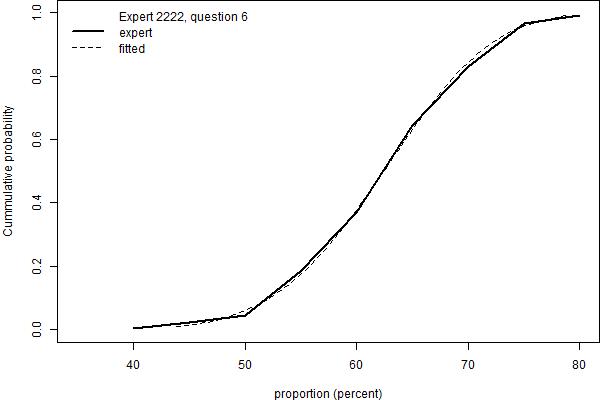 |
| Expert 3333  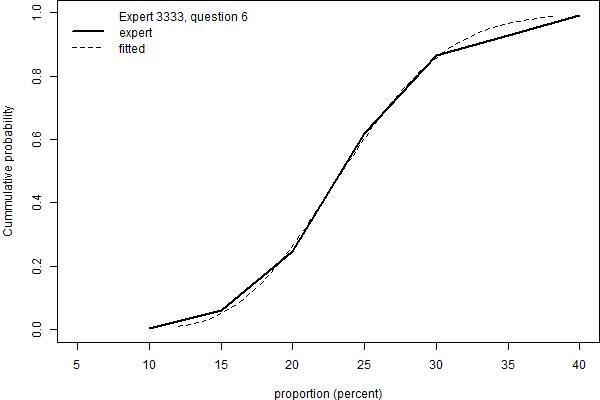 | Expert 4444  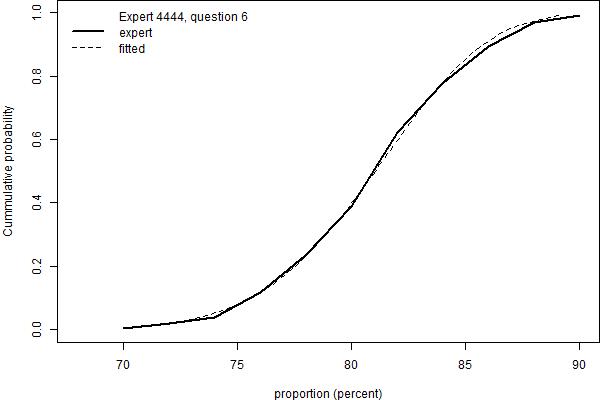 |
| **Question 7: Relapse and disease progression in previous year on moderately effective DMTs** | |
| Expert 1111  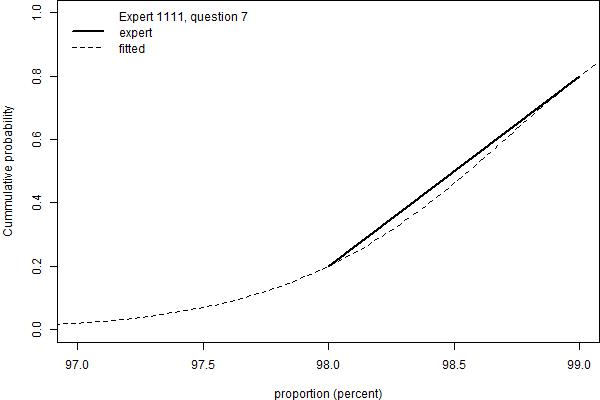 | Expert 2222  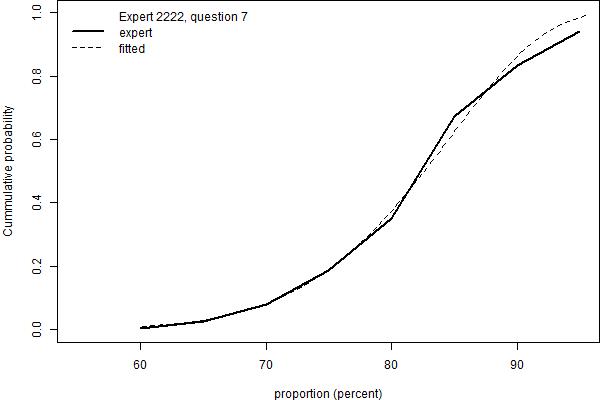 |
| Expert 3333  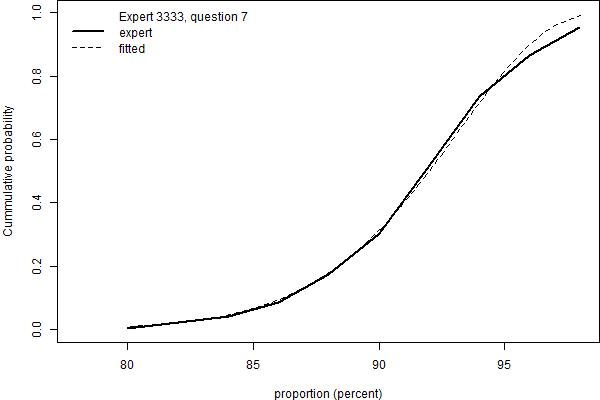 | Expert 4444  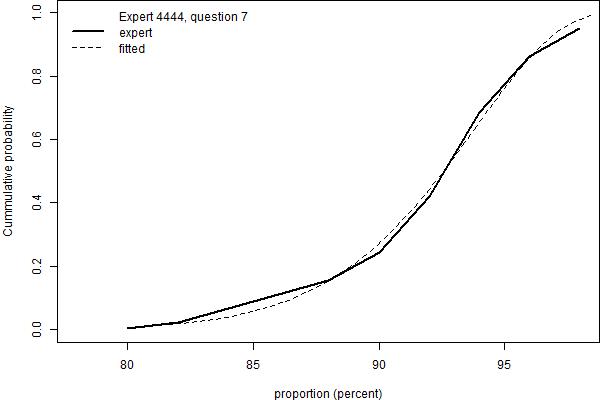 |
| **Question 8: Minimum age considering stop DMT treatment when stable disease** | |
| Expert 1111  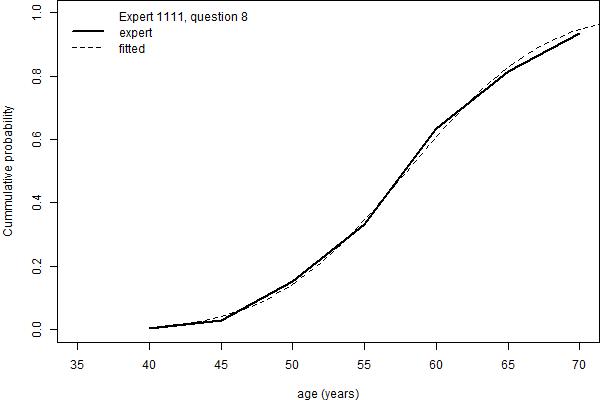 | Expert 2222  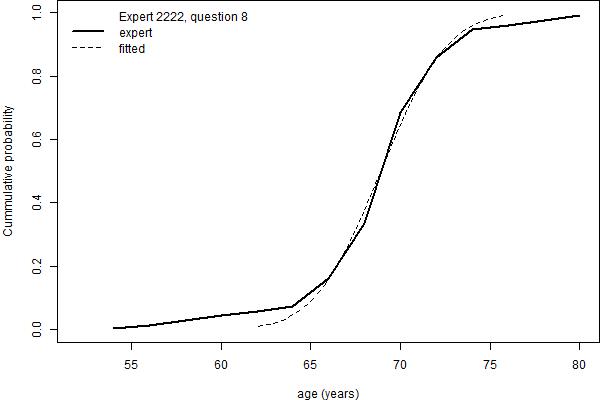 |
| Expert 3333  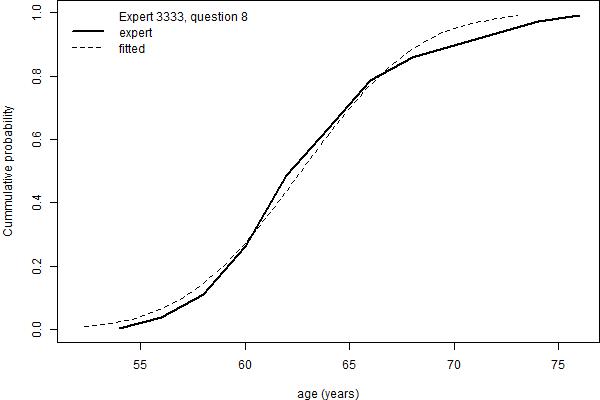 | Expert 4444  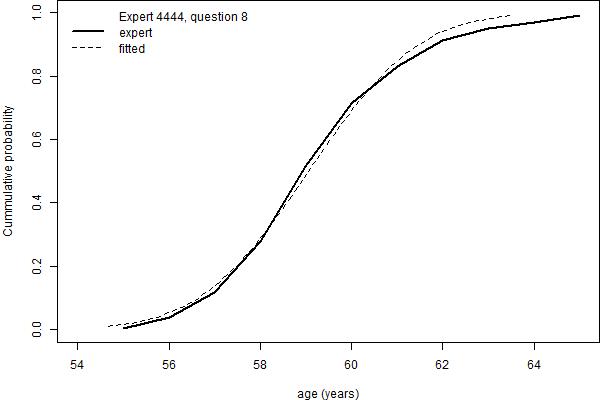 |
| **Question 9: Minimum duration of stable disease to consider stopping DMT treatment** | |
| Expert 1111  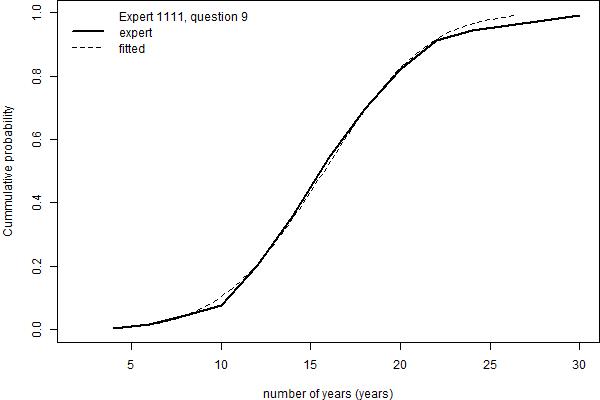 | Expert 2222  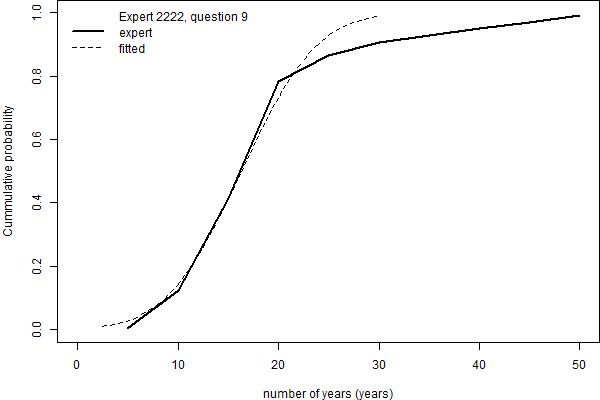 |
| Expert 3333  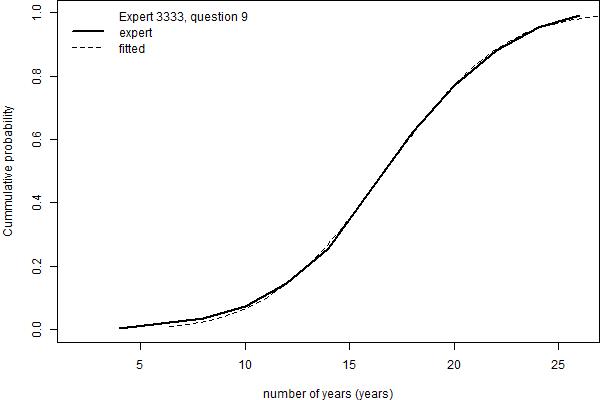 | Expert 4444  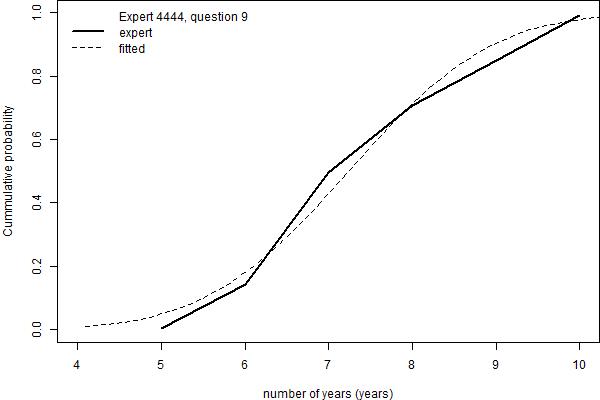 |

Supplemental figure 7. Observed versus fitted distributions for each individual expert and question. NB. Question 1 was a practice question and therefore not included.

| 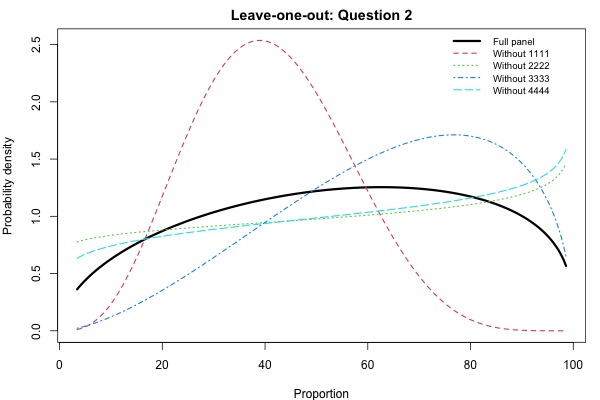 | 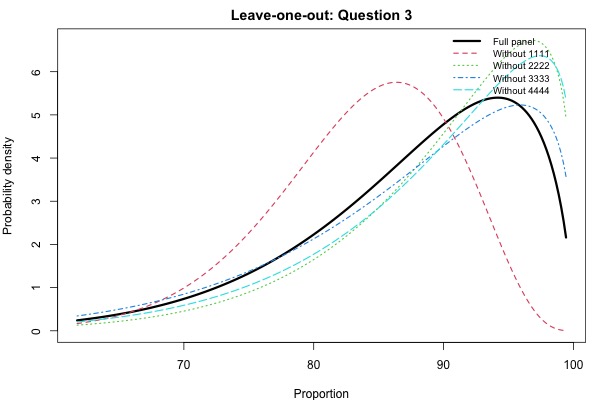 |
| --- | --- |
| 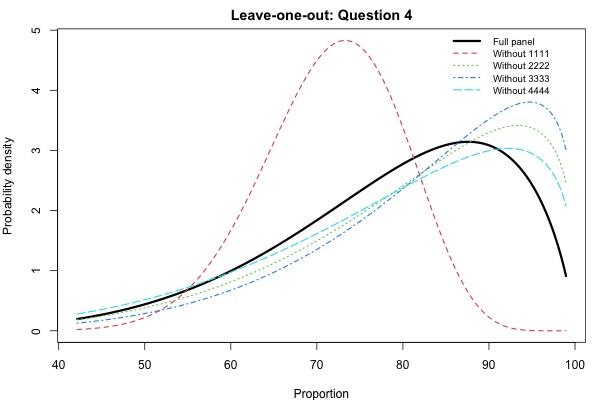 | 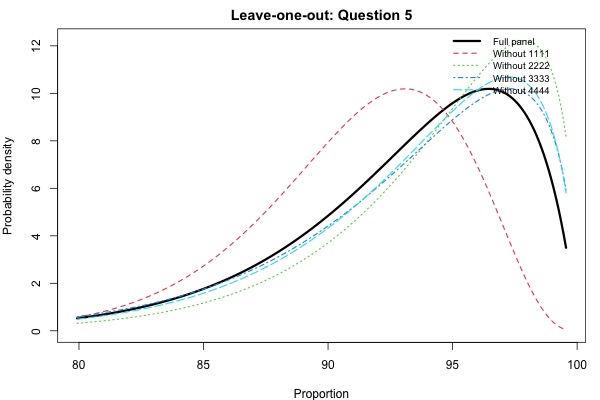 |
| 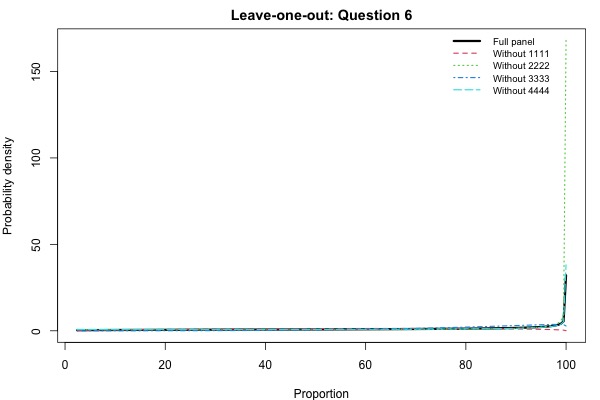 | 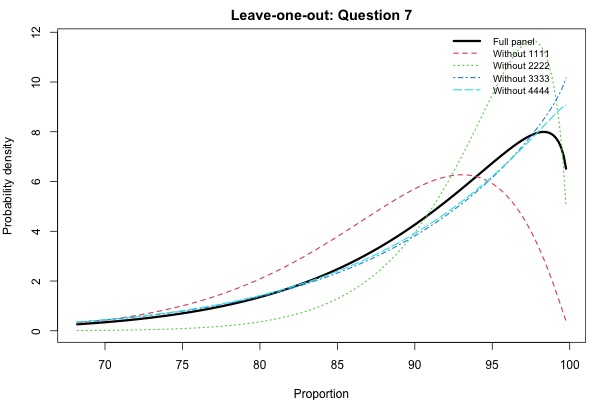 |
| 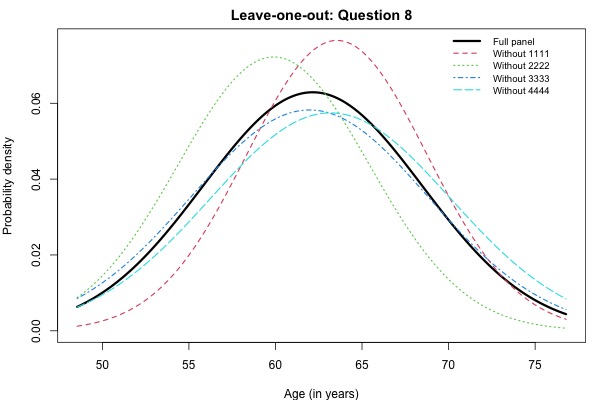 | 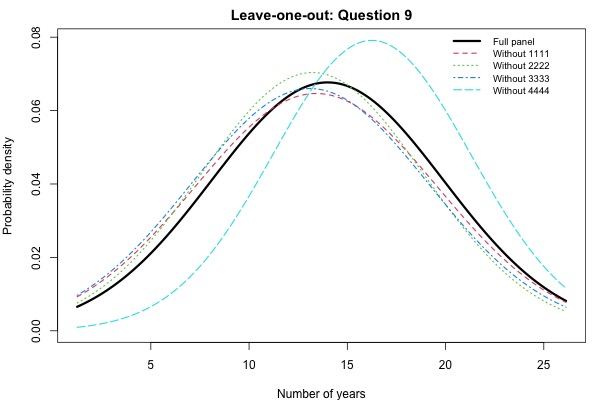 |

Supplemental figure 8. Distribution of outcomes of the leave-one-out analysis versus full expert panel.

NB. Question 1 was a practice question and therefore not included. Question 2: Relapse in previous year on highly effective DMTs, Question 3: Relapse in previous year on moderately effective DMTs, Question 4: Relapse in two subsequent years on highly effective DMTs, Question 5: Relapse in two subsequent years on moderately effective DMTs, Question 6: Relapse and disease progression in previous year on highly effective DMTs, Question 7: Relapse and disease progression in previous year on moderately effective DMTs, Question 8: Minimum age considering stop DMT treatment when stable disease, Question 9: Minimum duration of stable disease to consider stopping DMT treatment.


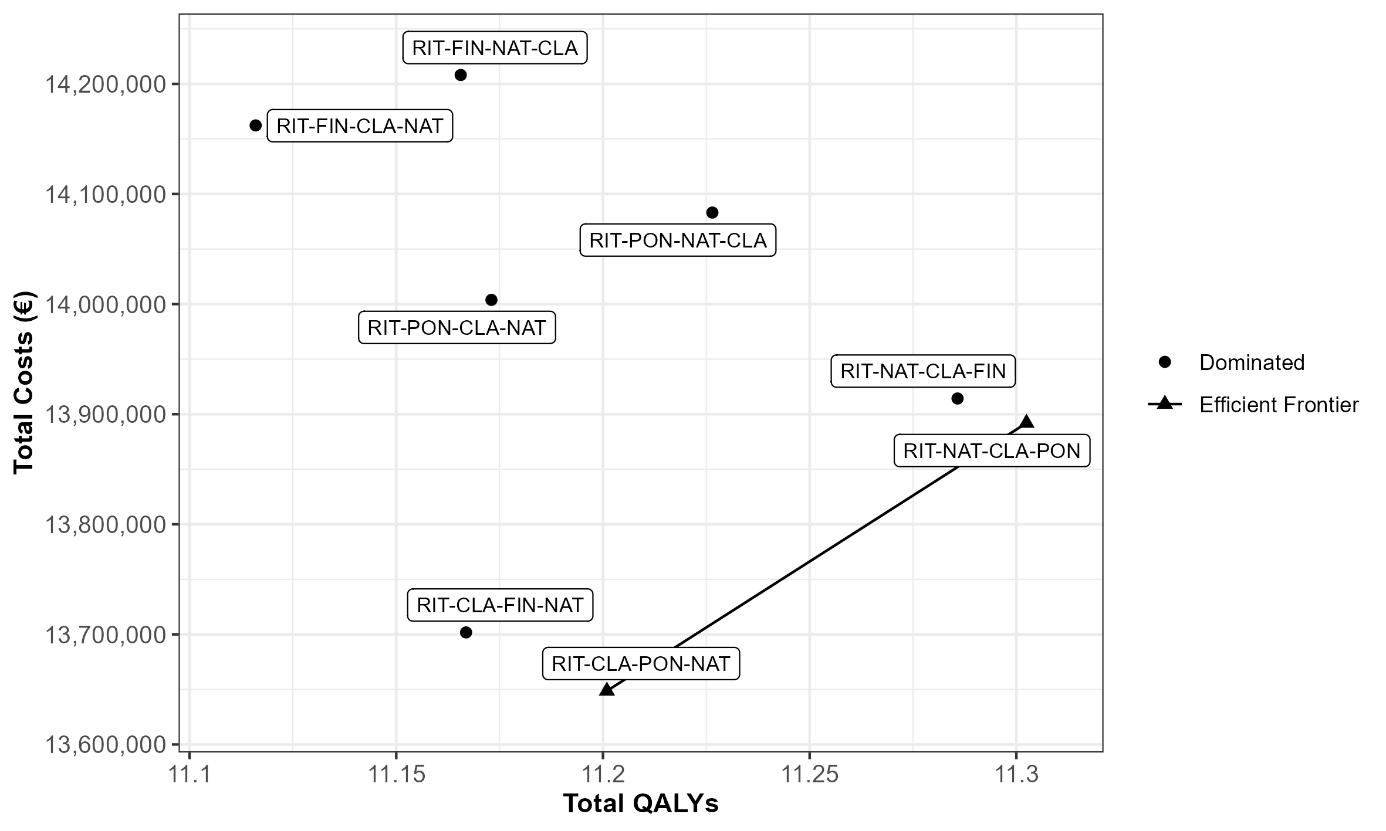


Supplemental figure 9. Efficiency frontier of the cost-effectiveness analysis based on probabilistic sensitivity analysis.
